# Recycling endosomal CD133 functions as an inhibitor of autophagy at the pericentrosomal region

**DOI:** 10.1038/s41598-019-39229-8

**Published:** 2019-02-19

**Authors:** Hideki Izumi, Yuanyuan Li, Masami Shibaki, Daisuke Mori, Michio Yasunami, Seiji Sato, Hisashi Matsunaga, Takao Mae, Kenji Kodama, Takehiko Kamijo, Yasuhiko Kaneko, Akira Nakagawara

**Affiliations:** 1 Laboratory of Molecular Medicine, Life Sciences Institute, Saga Medical Center KOSEIKAN, Saga, 840-8571 Japan; 2 Department of Laboratory Medicine, Saga Medical Center KOSEIKAN, Saga, Japan; 3 Saga Medical Center KOSEIKAN, Saga, 840-8571 Japan; 4 Research Institute for Clinical Oncology, Saitama Cancer Center, Saitama, 362-0806 Japan; 5 Saga HIMAT, Tosu, 841-0071 Japan

## Abstract

CD133 is a transmembranous protein that mainly localises to the plasma membrane in haematopoietic and neural stem cells as well as cancer stem cells. Although CD133 also localises to the cytoplasm, the mechanism of action and function of cytoplasmic CD133 currently remain unknown. We herein demonstrated that when Src family kinase activity is weak, CD133 interacts with HDAC6 and is transported to the pericentrosomal region after internalization and endosome formation via the dynein-based traffic system. Pericentrosomal CD133 is then recycled to the plasma membrane via recycling endosomes. At the pericentrosomal region, endosomal CD133 captures GABARAP, an initiator of autophagy, and inhibits GABARAP-mediated ULK1 activation and the subsequent initiation of autophagy. Furthermore, pericentrosomal CD133 suppresses cell differentiation, such as primary cilium formation and neurite outgrowth, by inhibiting autophagy. Thus, the present results provide evidence to suggest that pericentrosomal CD133 has the unique property of maintaining the undifferentiated status of cells by inhibiting autophagy.

## Introduction

CD133, also called prominin 1, was originally identified as a cell surface marker of human haematopoietic stem cells and mouse neuroepithelial cells^1–3^. It was subsequently reported to function as a marker of cancer stem cells in solid tumours, such as brain tumours^4^, colon cancer^5,6^, and hepatocellular carcinoma (HCC)^7^. The CD133-positive cell population has a greater self-renewal ability and chemoresistance phenotype than the CD133-negative cell population. The expression of CD133 correlates with malignant characteristics and a poor prognosis in many tumours^8^. CD133 is a pentaspan transmembranous protein that not only undergoes glycosylation at high levels, but also binds to cholesterol^9^. CD133 is phosphorylated in its intracellular C-terminal domain by Src family tyrosine kinases^10^. As a result, it activates the p85 subunit of phosphoinositide 3-kinase (PI-3K) by binding, and PI-3K, in turn, activates downstream targets such as Akt, thereby promoting cell proliferation in glioma stem cells^11^. CD133 is stabilized by binding with histone deacetylase 6 (HDAC6), and enhances the transcriptional activity of β-catenin, resulting in the acceleration of cell growth and suppression of cell differentiation^12^. CD133 is also known to function as a cancer stem cell marker in many cancers including neuroblastoma. When the expression of CD133 is down-regulated in neuroblastoma cells, neural differentiation frequently occurs^13^. Thus, CD133 is not only associated with tumour cell growth, but also regulates cell differentiation.

Recent studies reported that CD133 is directly involved in the cell survival of glioma and HCC through its role in the regulation of autophagy^14,15^. Autophagy is a highly conserved protein/organelle degradation system that is responsible for the turnover of long-lived proteins and disposal of excess or damaged organelles in order to maintain cell homeostasis^16,17^. Severe growth conditions, such as low nutrient levels, activate the autophagy pathway. ULK1 is at the top of this cascade and activates the autophagy initiation complex, and elongation of the isolation membrane also occurs^17,18^. The isolation membrane subsequently closes and engulfs cytoplasmic constituents, forming an autophagosome. The autophagosome fuses with a lysosome, resulting in the complete degradation of the sequestered cytoplasmic components by lysosomal enzymes^16,19^. Although the underlying mechanisms currently remain unknown, CD133 appears to be preferentially processed in endosomes^9,20^, and it has been reported to directly participate in the autophagosome membrane fusion process, and ultimately undergoes lysosomal degradation in the cytoplasm in some nutrient-deprived microenvironments^14,15,21^.

Autophagy also appears to serve as a critical mechanism for stem cell properties^22^. Autophagic activity is necessary for cell differentiation in neural stem cells (NSCs). In NSCs, autophagic activity is up-regulated during cell differentiation^22,23^. When autophagic activities are blocked by inhibitor(s), neurogenesis markedly decreases. Ambra1 is an autophagy component, and neuronal differentiation was shown to be impaired in *Ambra1*-null mice^24^. Thus, autophagy plays a critical role in NSC differentiation. In addition, autophagy was found to be up-regulated during the early differentiation of mouse and human embryonic stem cells (ESCs)^23^. The deletion of *Atg5* or *Beclin 1* resulted in defective embryoid bodies in mouse ESCs^25^, suggesting a pivotal role for autophagy in early embryonic development^23^. Autophagic activity is also involved in primary ciliogenesis^26–28^. Primary cilia are sensory organelles and the key coordinators of signalling pathways during development and tissue homeostasis. Cilia typically form in the growth-resting phase of the cell cycle^29^. Therefore, primary cilia form in many normal cells, but not in malignant tumour cells^29^.

In order to clarify the functions of CD133, we herein examined the cell localisation of CD133 in various cancer and normal cell lines under nutrient and nutrient-starved conditions, and found that CD133 has a unique property for autophagic processes. Mechanistically, we demonstrate that when Src family tyrosine kinase activity is weak, non-phosphorylated CD133 combined with HDAC6 is transported to endosomes, and is preferentially recruited to the pericentrosomal region via the dynein-based traffic system. We also show that pericentrosomal CD133 captures GABARAP at centrosomes in order to inhibit GABARAP-mediated ULK1 activation, and the subsequent initiation of autophagy.

## Results

### CD133 is transported from the plasma membrane to the pericentrosomal region

**Figure 1:**
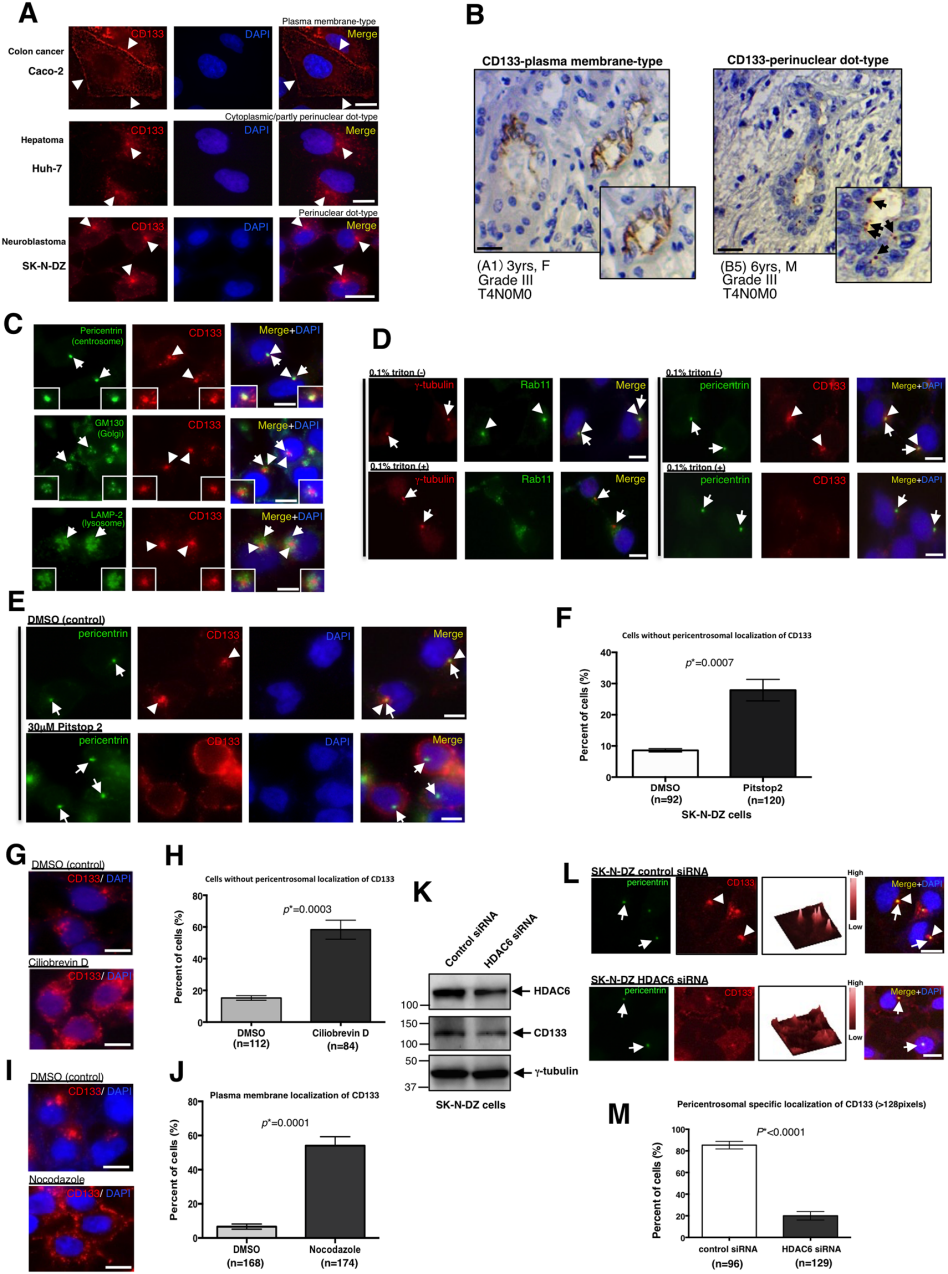
Membranous CD133 is transported to the pericentrosomal region via the dynein-based traffic system. (**A**) Representative immunostaining images of the subcellular localisation of CD133 in Caco-2, Huh-7, and SK-N-DZ cells. CD133 is red and DAPI (DNA) is blue. Arrowheads show the subcellular distribution of CD133. (**B**) Immunohistochemistry of CD133 in human neuroblastomas using a tissue microarray. Case (A1) shows the plasma membrane localisation of CD133. Case (B5) shows the perinuclear localisation of CD133. Insets show a magnified image. Arrows show the perinuclear localisation of CD133. Scale bars, 50 μm. (**C**) CD133 specifically localises to the pericentrosomal region, which is surrounded by the Golgi apparatus and lysosomes in SK-N-DZ cells. Pericentrin (centrosome), GM130 (Golgi), or LAMP-2 (lysosome) is green, CD133 is red, and DAPI (DNA) is blue. Arrows show centrosomes, the Golgi apparatus, or lysosomes. Arrowheads show CD133 signals. (**D**) The treatment with 0.1% Triton-X100 abolishes the pericentrosomal localisation of Rab11 and CD133, suggesting that CD133 localises to the pericentrosomal region as an endosome component. Representative immunostaining images of the subcellular localisation of Rab11 (left) and CD133 (right) in SK-N-DZ cells. γ-Tubulin (centrosome marker) is red, Rab11 is green, and DAPI (DNA) is blue (left). Pericentrin (centrosome marker) is green, CD133 is red, and DAPI (DNA) is blue (right). (**E**) CD133 is localised to the plasma membrane in SK-N-DZ cells after a treatment with Pitstop 2, an endosome inhibitor. Control cells were treated with 0.1% DMSO. Pericentrin is green, CD133 is red, and DAPI (DNA) is blue. (**F**) Quantification of cells without pericentrosomal localisation of CD133 in (**E**); mean ± standard error of the mean (SEM) from three experiments, *p = 0.0007. (**G**) A treatment with the selective cytoplasmic dynein inhibitor, ciliobrevin-D, causes the cell peripheral localisation near the plasma membrane of CD133 in SK-N-DZ cells. CD133 is red and DAPI (DNA) is blue. 0.1% DMSO-treated cells are used as a control. (**H**) Quantification of cells without pericentrosomal localisation of CD133 in (**G**); mean ± standard error of the mean (SEM) from three experiments, *p = 0.0003. (**I**) A treatment with the microtubule depolymerase, nocodazole, causes the plasma membrane localisation of CD133 in SK-N-DZ cells. CD133 is red and DAPI (DNA) is blue. 0.1% DMSO-treated cells are used as a control. (**J**) Quantification of cells with the plasma membrane localisation of CD133 in (**I**); mean ± standard error of the mean (SEM) from three experiments, *p = 0.0001. (**K**) Immunoblot of HDAC6 and CD133 expression in SK-N-DZ cells transfected with control siRNA or HDAC6 siRNA. An immunoblot of γ-tubulin served as a loading control. (**L**) Representative images of the disappearance of CD133 from the pericentrosomal region in SK-N-DZ cells transfected with control or HDAC6 siRNA. Pericentrin is green, CD133 is red, and DAPI (DNA) is blue. Arrows show centrosomes. Arrowheads show the pericentrosomal localisation of CD133. The signal intensity of CD133 in each cell is also shown as a three-dimensional figure. (**M**) Quantification of cells with the pericentrosomal localisation (>128 pixels) of CD133 in (**L**); mean ± standard error of the mean (SEM) from three experiments, *p < 0.0001. All scale bars, 10 μm.

CD133 is a pentaspan transmembrane protein. However, a recent study showed that CD133 localises around the cytoplasm in many tumours^14,30,31^. Therefore, we investigated the localisation status of CD133 in CD133-positive cancer cell lines using immunostaining (Fig. 1A). While CD133 localised to the plasma membrane in Caco-2 cells, it mainly localised around the cytoplasm and partly to the perinuclear region in Huh-7 cells (Fig. 1A) as a dot-like structure. Moreover, CD133 also specifically localised to the perinuclear region as a dot-like structure in SK-N-DZ cells (Fig. 1A). We also investigated the localisation status of CD133 in these cancer cell lines using other anti-CD133 antibodies, and similar results were obtained (Supplementary Fig. S1A). We then performed immunohistochemistry experiments using human neuroblastoma clinical samples (Fig. 1B). Two out of 25 human neuroblastoma samples were CD133-positive. While CD133 mainly localised to the plasma membrane in one case (Fig. 1B, left), it specifically localised to the perinuclear region as a dot-like structure in another case (Fig. 1B, right). We also examined the intracellular localisation of CD133 by immunostaining using pericentrin (centrosome), GM130 (Golgi), and LAMP2 (lysosome) antibodies. The results obtained revealed that CD133 specifically localised to the pericentrosomal region, which is surrounded by the Golgi apparatus and lysosomes, in SK-N-DZ cells (Fig. 1C; and Supplementary Fig. S1B).

Since CD133 was originally identified as a transmembrane protein, we speculated that endosomal CD133 is recruited to the pericentrosomal region. In order to confirm this, we attempted to perform an immunostaining experiment using 0.1% Triton X-100 extraction prior to fixation because Triton X-100 extraction is known to exclude soluble components from cells^32,33^. When the signal of Rab11, an endosome marker, was eliminated using this extraction (Fig. 1D, left), the pericentrosomal CD133 signal was also eliminated (Fig. 1D, right). However, centrosome components such as γ-tubulin and pericentrin were resistant to Triton X-100 extraction (Fig. 1D). These results suggested that endosomal CD133 localises to the pericentrosomal region. In order to support these results, we used Pitstop 2, a chemical compound that inhibits clathrin-dependent endocytosis^34^. The Pitstop 2 treatment significantly induced the plasma membrane localisation of CD133, but not pericentrosomal localisation in SK-N-DZ and Huh-7 cells (Fig. 1E,F; Supplementary Fig. S1C,D). These results suggest that CD133 initially localises to the plasma membrane and is then rapidly transported to the pericentrosomal region via endocytosis. After endocytosis, dynein motors assist endosome trafficking along microtubules. Therefore, we disrupted this trafficking process using the cytoplasmic dynein inhibitor, ciliobrevin D^35^, and microtubule disassembly drug, nocodazole. As expected, the endosomal CD133 signal was detected at the cell periphery and/or plasma membrane, but not in the pericentrosomal region following the treatments with ciliobrevin D and nocodazole, respectively (Fig. 1G–J). Therefore, endosomal CD133 was recruited to the pericentrosomal region via the dynein-based traffic system.

Since the CD133 protein is stabilized by HDAC6 binding^12^ and HDAC6 also interacts with dynein^36^, we performed a partial knockdown experiment using HDAC6 siRNA because the complete knockdown of HDAC6 strongly induces protein instability in CD133^12^. When HDAC6 expression was partially knocked down (Fig. 1K), the pericentrosomal CD133 signal was partly abolished (Fig. 1L,M). The CD133 signal was instead detected in the cell periphery. Since HDAC6 interacts with CD133 at the pericentrosomal region in SK-N-DZ cells (Supplementary Fig. S1E,F), this result suggested that HDAC6 not only stabilizes the CD133 protein, but is also involved in CD133 trafficking to the pericentrosomal region.

### CD133-positive endosomes are recycling endosomes

**Figure 2:**
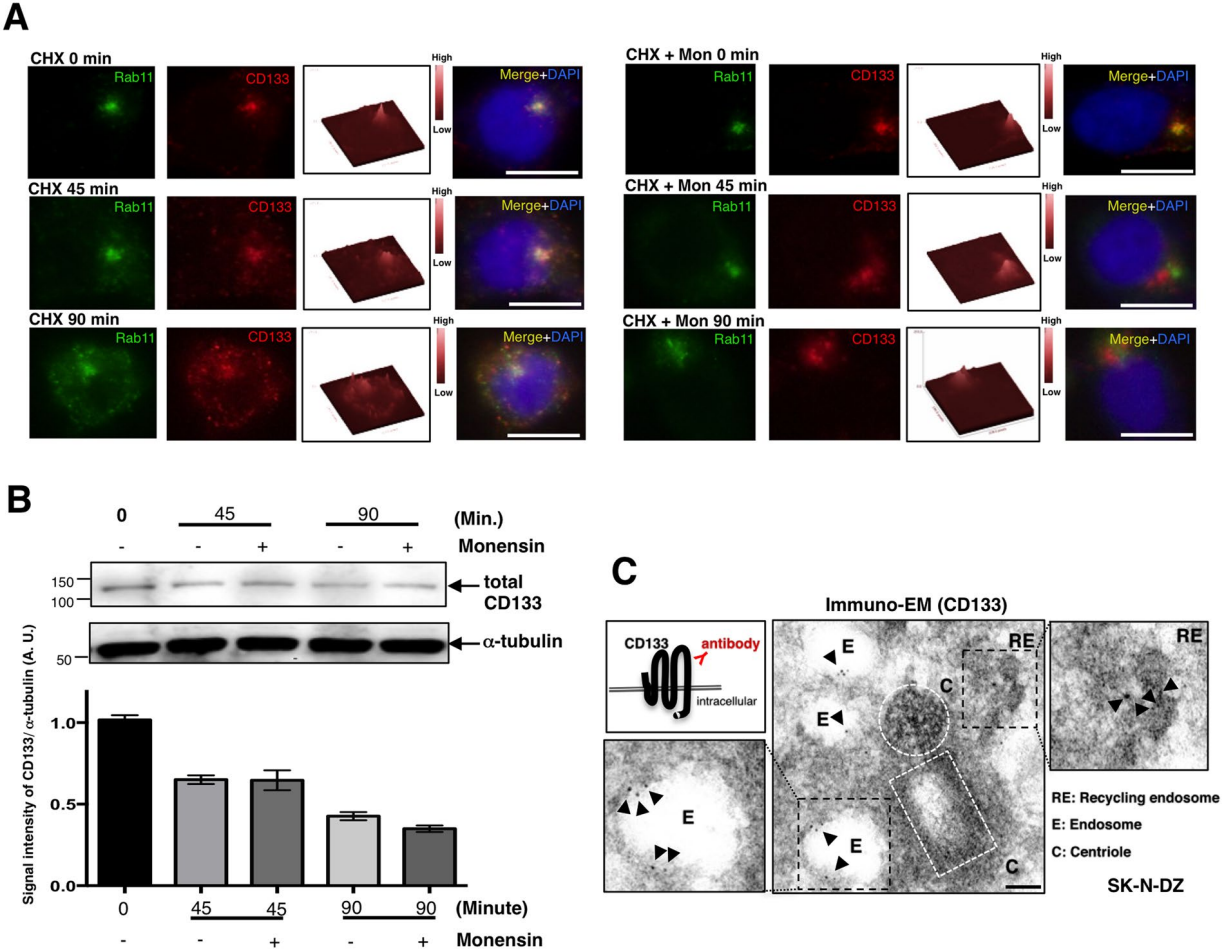
CD133-positive endosomes are recycling endosomes. (**A**) Representative images of CD133 dissociated from the pericentrosomal region in SK-N-DZ cells treated with cycloheximide (CHX) or CHX plus monensin (Mon) for 90 min. Rab11 is green, CD133 is red, and DAPI (DNA) is blue. The CD133 signal appeared along the plasma membrane (left). On the other hand, when protein trafficking was blocked by monensin (Mon), the CD133 signal remained at the pericentrosomal region (right). The signal intensity of CD133 in each cell is also shown as a three-dimensional figure. Scale bars, 10 μm. (**B**) Immunoblot of total CD133 expression in cells treated with CHX for 0, 45, and 90 min with or without the monensin treatment. The quantification of CD133 band expression intensities is also shown as a graph (±standard error of the mean (SEM) from three experiments). An immunoblot of α-tubulin served as a loading control. (**C**) Immune electron micrograph of CD133 in a SK-N-DZ cell. Immunogold CD133 signals are shown by arrowheads. The white dotted lines show centrioles. Scale bars, 200 nm.

Since the pericentrosomal CD133 signal is coincident with the Rab11-containing recycling endosome signal (Fig. 2A, upper panel (CHX 0 min)), we investigated whether CD133-positive endosomes are recycling endosomes. When new protein synthesis was blocked by a cycloheximide (CHX) treatment for 90 min, CD133 signals appeared along the plasma membrane (Fig. 2A, left). In addition, when protein trafficking was blocked by the ionophore, monensin, as reported previously^37^, CD133 signals were retained at the pericentrosomal region (Fig. 2A, right). Since monensin inhibits endosome-to-plasma membrane recycling^37^, the accumulation of CD133 occurred in a pericentrosomal region when new protein synthesis was also inhibited (Fig. 2A,B, right). Therefore, we concluded that intracellular CD133 is located in recycling endosomes at the pericentrosomal region. We also confirmed that pericentrosomal CD133 is located in these endosomes using immune electron microscopy (Fig. 2C). As expected, the immunogold CD133 signal was detected in endosomes and recycling endosomes near the centrioles.

### Src activity is necessary for the plasma membrane localisation of CD133

**Figure 3:**
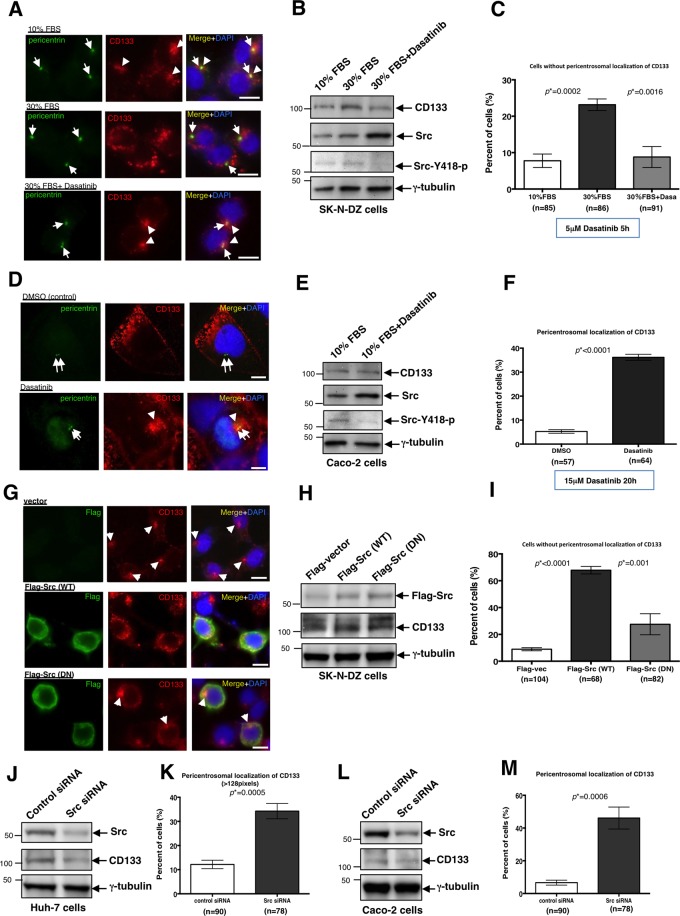
Src activity is necessary for the plasma membrane localisation of CD133. (**A**) Representative images of CD133 localisation in SK-N-DZ cells with 10% FBS, 30% FBS, and 30% FBS plus the Src/Abl inhibitor, dasatinib. Pericentrin is green, CD133 is red, and DAPI (DNA) is blue. Arrows show centrosomes. Arrowheads show CD133 signals at the pericentrosomal region. (**B**) Immunoblot of CD133, Src, and Src-Y418-p in SK-N-DZ cells with the treatment indicated above each lane. An immunoblot of γ-tubulin served as a loading control. (**C**) Quantification of SK-N-DZ cells without pericentrosomal localisation of CD133 shown in (**A**); mean ± standard error of the mean (SEM) from three experiments, *p = 0.0002, *p = 0.0016, respectively. (**D**) Representative images of CD133 signals localised at the pericentrosomal region in Caco-2 cells treated with dasatinib. 0.1% DMSO-treated cells are used as a control. Pericentrin is green, CD133 is red, and DAPI (DNA) is blue. Arrows show centrosomes. Arrowheads show CD133 signals at the pericentrosomal region. (**E**) Immunoblot of CD133, Src, and Src-Y418-p in Caco-2 cells treated as indicated above each lane. An immunoblot of γ-tubulin served as a loading control. (**F**) Quantification of cells with the pericentrosomal localisation of CD133 in (**D**); mean ± standard error of the mean (SEM) from three experiments, *p < 0.0001. In this experiment, peripheral colony-forming Caco-2 cells were counted. (**G**) Representative images of CD133 localisation in SK-N-DZ cells transfected with the control vector, Flag-Src (wild type (WT)), and Flag-Src (dominant negative (DN)). Flag is green, CD133 is red, and DAPI (DNA) is blue. Arrowheads show CD133 signals at the pericentrosomal region. (**H**) Immunoblot of CD133 and Flag in SK-N-DZ cells transfected with the vector indicated above each lane. An immunoblot of γ-tubulin served as a loading control. (**I**) Quantification of cells without pericentrosomal localisation of CD133 shown in (**G**); mean ± standard error of the mean (SEM) from three experiments, *p < 0.0001, *p = 0.001, respectively. (**J**) Immunoblot of Src and CD133 in Huh-7 cells transfected with control or Src siRNA. An immunoblot of γ-tubulin served as a loading control. (**K**) Quantification of cells with CD133 signals (>128 pixels) at the pericentrosomal region used in the experiment in (**J**); mean ± standard error of the mean (SEM) from three experiments, *p = 0.0005. (**L**) Immunoblot of Src and CD133 in Caco-2 cells transfected with control or Src siRNA. An immunoblot of γ-tubulin served as a loading control. (**M**) Quantification of cells with CD133 signals at the pericentrosomal region used in the experiment in (**L**); mean ± standard error of the mean (SEM) from three experiments, *p = 0.0006. In this experiment, peripheral colony-forming Caco-2 cells were counted. All scale bars, 10 μm.

We investigated the relationship between Src kinase and CD133 because Src kinase is known to phosphorylate CD133^10^. When Src activity was up-regulated in growth medium with a high concentration (30%) of FBS, CD133 localised in the cell periphery near the plasma membrane in SK-N-DZ cells (Fig. 3A,C). However, when the Src inhibitor, dasatinib was added to growth medium containing 30% FBS, CD133 was translocated to its pericentrosomal localisation (Fig. 3A–C). This effect was observed not only in SK-N-DZ cells, but also in Caco-2 cells. When we added dasatinib to Caco-2 cells in culture, CD133 localised to the pericentrosomal region (Fig. 3D–F). These results indicated that Src activity is necessary for the localisation of CD133 to the plasma membrane. When Flag tagged-Src was transfected into SK-N-DZ cells, CD133 changed its pericentrosomal localisation to the cell peripheral cytoplasm (Fig. 3G–I). However, when the dominant-negative (DN) type of Flag-tagged Src was transfected into SK-N-DZ cells, CD133 remained in the pericentrosomal region (Fig. 3G–I). In addition, the knockdown of Src expression significantly increased the pericentrosomal localisation of CD133 in Huh-7 and Caco-2 cells (Fig. 3J–M and Supplementary Fig. S2). These results indicated that Src-mediated phosphorylation maintains the localisation of CD133 at the plasma membrane.

### Phosphorylation status of CD133 influences its subcellular localisation

**Figure 4:**
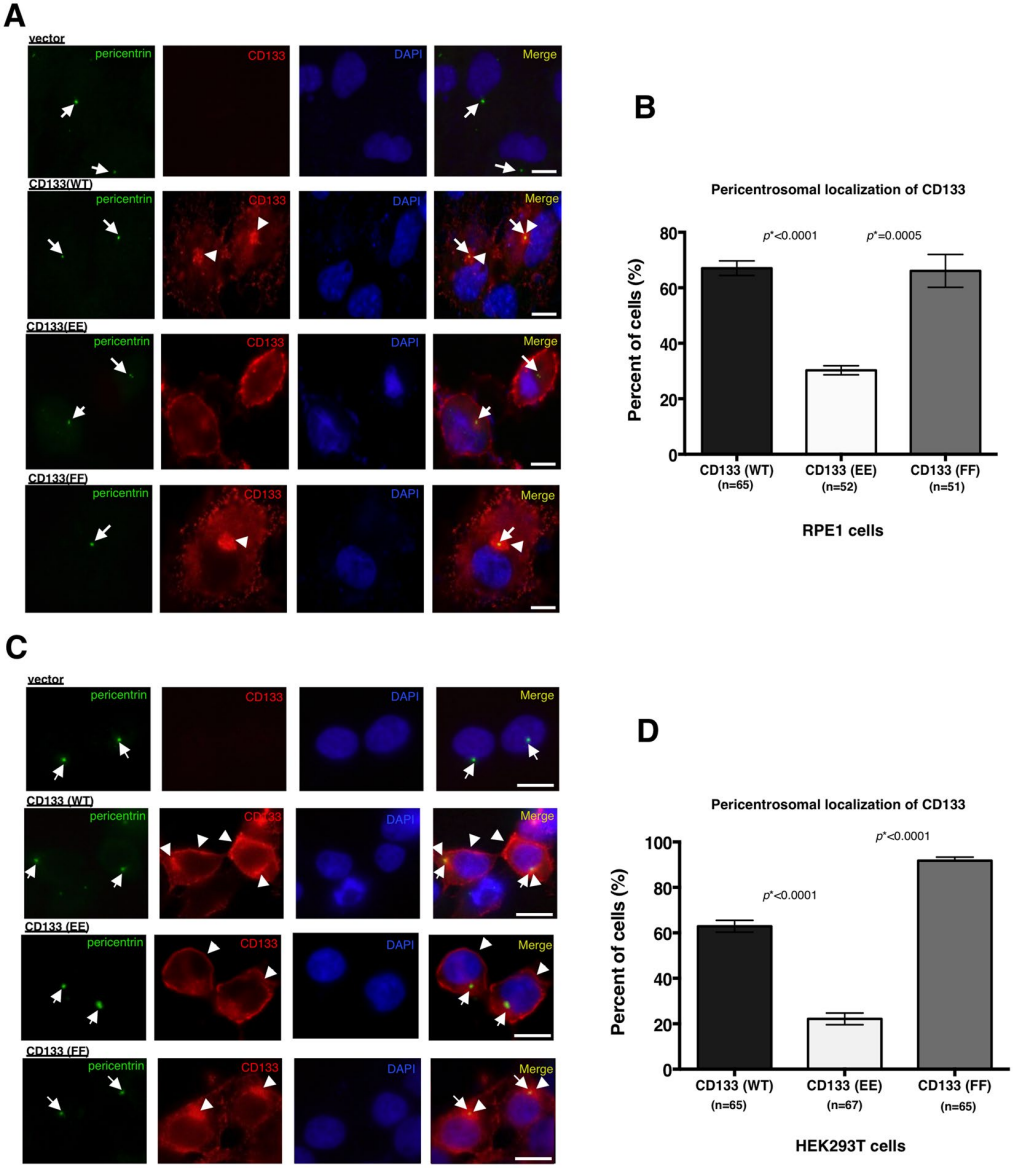
The phosphorylation status of CD133 affects its subcellular localisation. (**A**) Representative images of CD133 localisation in human normal RPE1 cells transfected with one of the following four vectors: empty vector (vector), CD133 (wild-type (WT)), CD133 (phospho-mimic (EE)), and CD133 (phospho-deficient (FF)). Pericentrin is green, CD133 is red, and DAPI (DNA) is blue. Arrows show centrosomes. Arrowheads show CD133 signals localised to the pericentrosomal region. (**B**) Quantification of RPE1 cells with CD133 signals at the pericentrosomal region shown in (**A**); mean ± standard error of the mean (SEM) from three experiments, *p < 0.0001, *p = 0.0005, respectively. (**C**) Representative images of CD133 localisation in HEK293T cells transfected with one of the following four vectors: empty vector (vector), CD133 (wild-type (WT)), CD133 (phospho-mimic (EE)), and CD133 (phospho-deficient (FF)). Pericentrin is green, CD133 is red, and DAPI (DNA) is blue. Arrows show centrosomes. Arrowheads show CD133 signals localised to the pericentrosomal and/or plasma membrane regions. (**D**) Quantification of cells with CD133 signals at the pericentrosomal region shown in (**C**); mean ± standard error of the mean (SEM) from three experiments, *p < 0.0001, *p < 0.0001, respectively. All scale bars, 10 μm.

Since CD133 has two Src phosphorylation sites (Y828 and Y852)^10^, we constructed two expression vectors: the phosphorylation-mimic CD133 (CD133 (EE)) and phosphorylation-deficient mutant of CD133 (CD133 (FF)), and then transfected them into non-tumoural human cells, such as TERT-mediated immortalized retina pigment epithelial 1 (RPE1) cells and human embryonic kidney 293 T (HEK293T) cells, which do not express endogenous CD133. While CD133 (EE) dominantly localised to the plasma membrane, wild-type CD133 (CD133 (WT)) and CD133 (FF) both mainly localised to the pericentrosomal region in RPE1 cells (Fig. 4A,B). We subsequently performed the same experiment using HEK293T cells. In this case, we obtained similar results to the RPE1 cell experiment (Fig. 4C,D). Since human non-tumoural cells generally exhibit weak Src kinase activity, CD133 may be preferentially processed in endosomes, and endosomal CD133 may then be transported to the pericentrosomal region in normal cells.

### Pericentrosomal CD133 suppresses the activation of autophagy due to the accumulation of GABARAP at centrosomes

**Figure 5:**
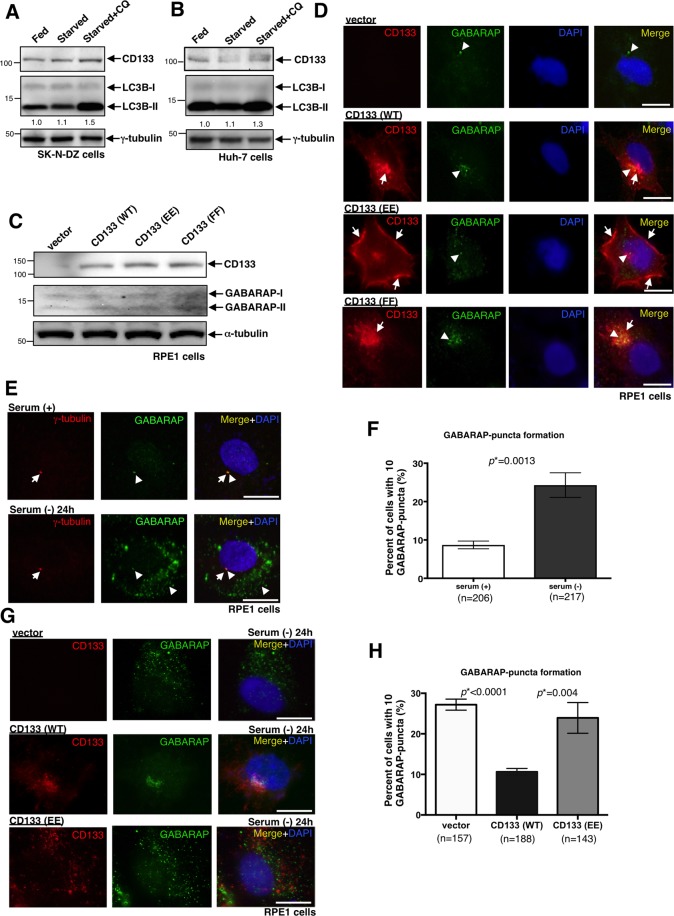
Pericentrosomal CD133 suppresses the activation of autophagy due to the centrosomal accumulation of GABARAP. (**A**) Immunoblot of CD133 and LC3B in SK-N-DZ cells with or without serum starvation plus chloroquine (CQ) for 20 h. An immunoblot of γ-tubulin served as a loading control. LC3B-II/γ-tubulin ratios are shown between the 2 blots in numerical values. (**B**) Immunoblot of CD133 and LC3B in Huh-7 cells with or without serum starvation plus CQ for 20 h. An immunoblot of γ-tubulin served as a loading control. LC3B-II/γ-tubulin ratios are shown between the 2 blots in numerical values. (**C**) Immunoblot of CD133 and GABARAP in RPE1 cells transfected with one of the following four vectors: empty vector (vector), CD133 (WT), CD133 (EE), or CD133 (FF). An immunoblot of α-tubulin served as a loading control. (**D**) Representative images of CD133 and GABARAP signals localised to the pericentrosomal or membranous region in RPE1 cells transfected with one of the following four vectors: empty vector (vector), CD133 (WT), CD133 (EE), and CD133 (FF). CD133 is red, GABARAP is green, and DAPI (DNA) is blue. Arrows show centrosomes or the plasma membrane. Arrowheads show the localisation pattern of GABARAP. Scale bars, 10 μm. (**E**) Representative images of GABARAP signals localised to the centrosome or cytoplasm in RPE1 cells with or without serum starvation for 24 h. γ-Tubulin is red, GABARAP is green, and DAPI (DNA) is blue. Arrows show centrosomes. Arrowheads show the centrosomal localisation and cytoplasmic puncta of GABARAP. Scale bars, 10 μm. (**F**) Quantification of cells with GABARAP-puncta shown in (**E**); mean ± standard error of the mean (SEM) from three experiments, *p = 0.0013. (**G**) Representative images of GABARAP-puncta in RPE1 cells transfected with empty vector (vector), CD133 (WT), or CD133 (EE) expression vector, followed by serum starvation for 24 h. CD133 is red, GABARAP is green, and DAPI (DNA) is blue. Scale bars, 10 μm. (**H**) Quantification of cells with GABARAP-puncta shown in (**G**); mean ± standard error of the mean (SEM) from three experiments, *p < 0.0001, *p = 0.004, respectively.

Previous studies reported that CD133 is involved in the regulation of autophagy^14,15,21^. Therefore, we initially investigated autophagic activity in two pericentrosomal CD133-positive cell lines, SK-N-DZ and Huh-7 cells by immunoblotting using the LC3B antibody. Theses two cell lines showed low autophagy induction abilities even when serum starvation was conducted for 24 h (Fig. 5A,B). Thus, we hypothesized that centrosome-resident CD133 may suppress autophagy. We investigated whether pericentrosomal CD133 affects GABARAP localisation because this protein has been identified as a centrosome-resident autophagy mediator that initiates the activation of ULK1 kinase by its binding^38,39^. We transfected CD133 expression vectors into RPE1 cells (Fig. 5C), and then examined GABARAP localisation (Fig. 5D). We found that GABARAP accumulated in the centrosomes of CD133 (WT)- and CD133 (FF)-transfected cells (Fig. 5D), but not CD133 (EE)-transfected cells (Fig. 5D). The accumulation of GABARAP in centrosomes did not appear to be due to the up-regulated expression of GABARAP because the expression level of endogenous GABARAP was very low and did not change in RPE1 cells transfected with one of the 3 types of CD133 expression vectors (Fig. 5C).

When serum starvation was conducted for 24 h to induce autophagy, RPE1 cells showed many GABARAP-puncta in the cytoplasm (Fig. 5E,F). Therefore, we investigated the frequency of GABARAP-puncta formation in CD133 transfected RPE1 cells when serum was starved for 24 h. We found that while a high frequency of GABARAP-puncta was detected in the vector- and CD133 (EE)-transfected cells (Fig. 5G,H), it was significantly suppressed in CD133 (WT)-transfected cells during serum starvation for 24 h (Fig. 5G,H). When we also employed SK-N-DZ cells in a similar serum starvation experiment, we found that while large amounts of GABARAP accumulated at centrosomes, GABARAP-puncta were rarely observed in the cytoplasm (Supplementary Fig. S3). These results suggested that pericentrosomal CD133 suppresses the activation of autophagy due to the accumulation of GABARAP at centrosomes.

### Knockdown of CD133 induces autophagy in pericentrosomal CD133-positive cells

**Figure 6:**
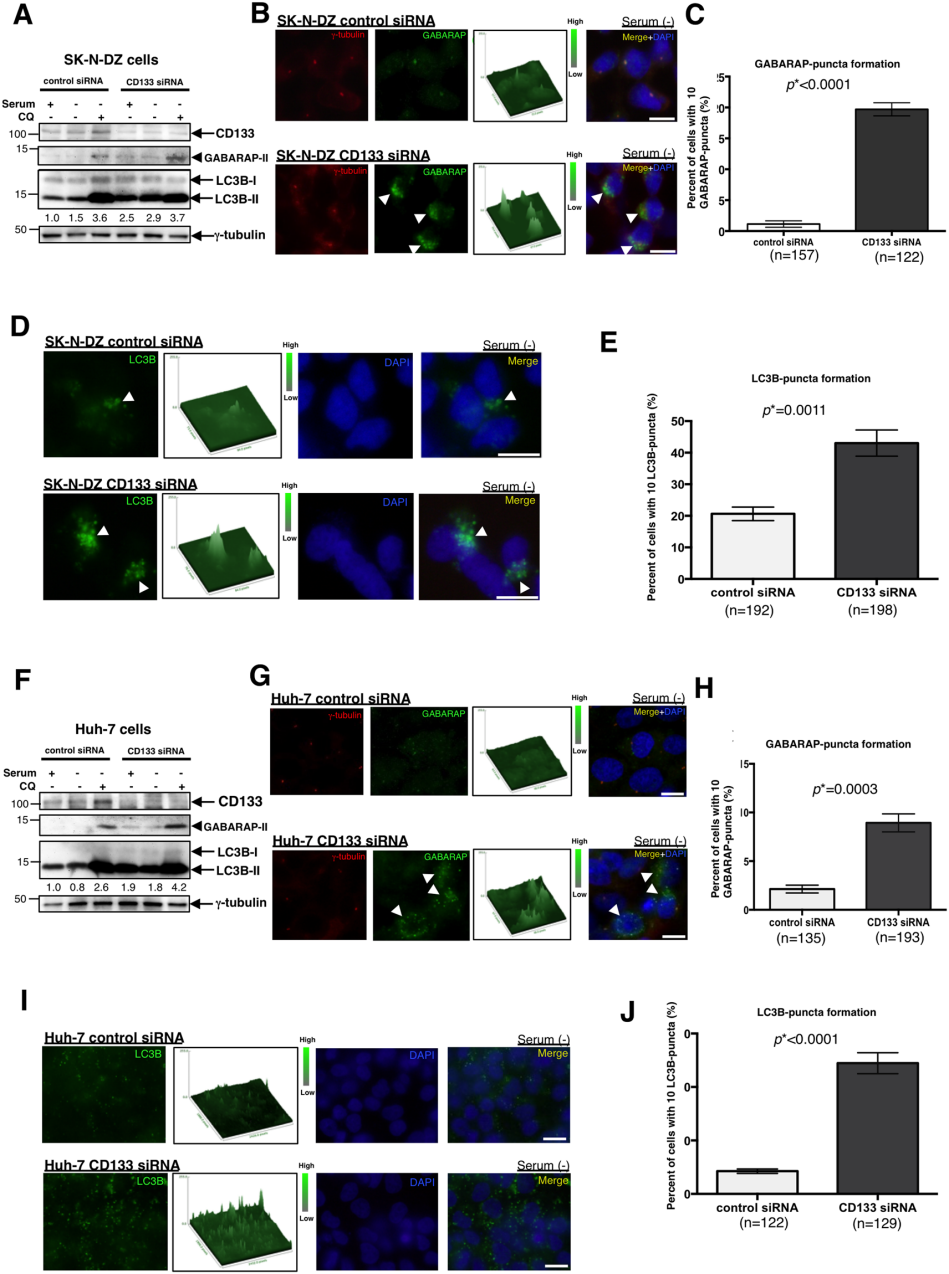
Knockdown of CD133 induces autophagy in SK-N-DZ and Huh-7 cells. (**A**) Immunoblot of CD133, GABARAP, and LC3B in SK-N-DZ cells transfected with control siRNA or CD133 siRNA with or without serum plus Chloroquine (CQ). An immunoblot of γ-tubulin served as a loading control. LC3B-II/γ-tubulin ratios are shown between the 2 blots in numerical values. (**B**) Representative images of GABARAP-puncta patterns in SK-N-DZ cells transfected with CD133 siRNA or its control, followed by serum starvation for 48 h. γ-Tubulin is red, GABARAP is green, and DAPI (DNA) is blue. Arrowheads show the cytoplasmic puncta of GABARAP. The signal intensity of GABARAP-puncta in each cell is also shown as a three-dimensional figure. (**C**) Quantification of cells with 10 GABARAP-puncta shown in (**B**); mean ± standard error of the mean (SEM) from three experiments, *p < 0.0001. (**D**) Representative images of LC3B-puncta in SK-N-DZ cells transfected with CD133 siRNA or its control, followed by serum starvation for 48 h. LC3B is green and DAPI (DNA) is blue. Arrowheads show the cytoplasmic puncta of LC3B. The signal intensity of LC3B-puncta in each cell is also shown as a three-dimensional figure. (**E**) Quantification of cells with 10 LC3B-puncta in (**D**); mean ± standard error of the mean (SEM) from three experiments, *p = 0.0011. (**F**) Immunoblot of CD133, GABARAP, and LC3B in Huh-7 cells transfected with CD133 siRNA or its control with or without serum plus CQ. An immunoblot of γ-tubulin served as a loading control. LC3B-II/γ-tubulin ratios are shown between the 2 blots in numerical values. (**G**) Representative images of GABARAP-puncta in Huh-7 cells transfected with CD133 siRNA or its control, followed by serum starvation for 48 h. γ-Tubulin is red, GABARAP is green, and DAPI (DNA) is blue. Arrowheads show the cytoplasmic puncta of GABARAP. The signal intensity of GABARAP-puncta in each cell is also shown as a three-dimensional figure. (**H**) Quantification of cells with 10 GABARAP-puncta shown in (**G**); mean ± standard error of the mean (SEM) from three experiments, *p = 0.0003. (**I**) Representative images of LC3B-puncta in Huh-7 cells transfected with CD133 siRNA or its control, followed by serum starvation for 48 h. LC3B is green and DAPI (DNA) is blue. The signal intensity of LC3B-puncta in each cell is also shown as a three-dimensional figure. (**J**) Quantification of cells with 10 LC3B-puncta shown in (**I**); mean ± standard error of the mean (SEM) from three experiments, *p < 0.0001. All scale bars, 10 μm.

Since pericentrosomal CD133 appears to suppress the activation of autophagy, the expression of CD133 was knocked down in SK-N-DZ. As expected, immunoblotting showed that GABARAP-II and LC3B-II band intensities increased in CD133-knockdown SK-N-DZ cells (Fig. 6A). Moreover, centrosomal GABARAP signals were weaker in CD133-knockdown SK-N-DZ cells than in control-transfected cells (Supplementary Fig. S4A,B). CD133-knockdown SK-N-DZ cells were then serum-starved for 48 h, and immunostaining experiments were performed. As expected, autophagy-related GABARAP- and LC3B- puncta significantly increased in CD133-knockdown SK-N-DZ cells (Fig. 6B–E Supplementary Fig. S4C,D). Similar results were obtained in CD133-knockdown Huh-7 cells (Fig. 6F–J). Therefore, we concluded that pericentrosomal CD133 inhibits the activation of autophagy.

### Pericentrosomal CD133 captures GABARAP at centrosomes, thereby inhibiting GABARAP-mediated ULK1 activation

**Figure 7:**
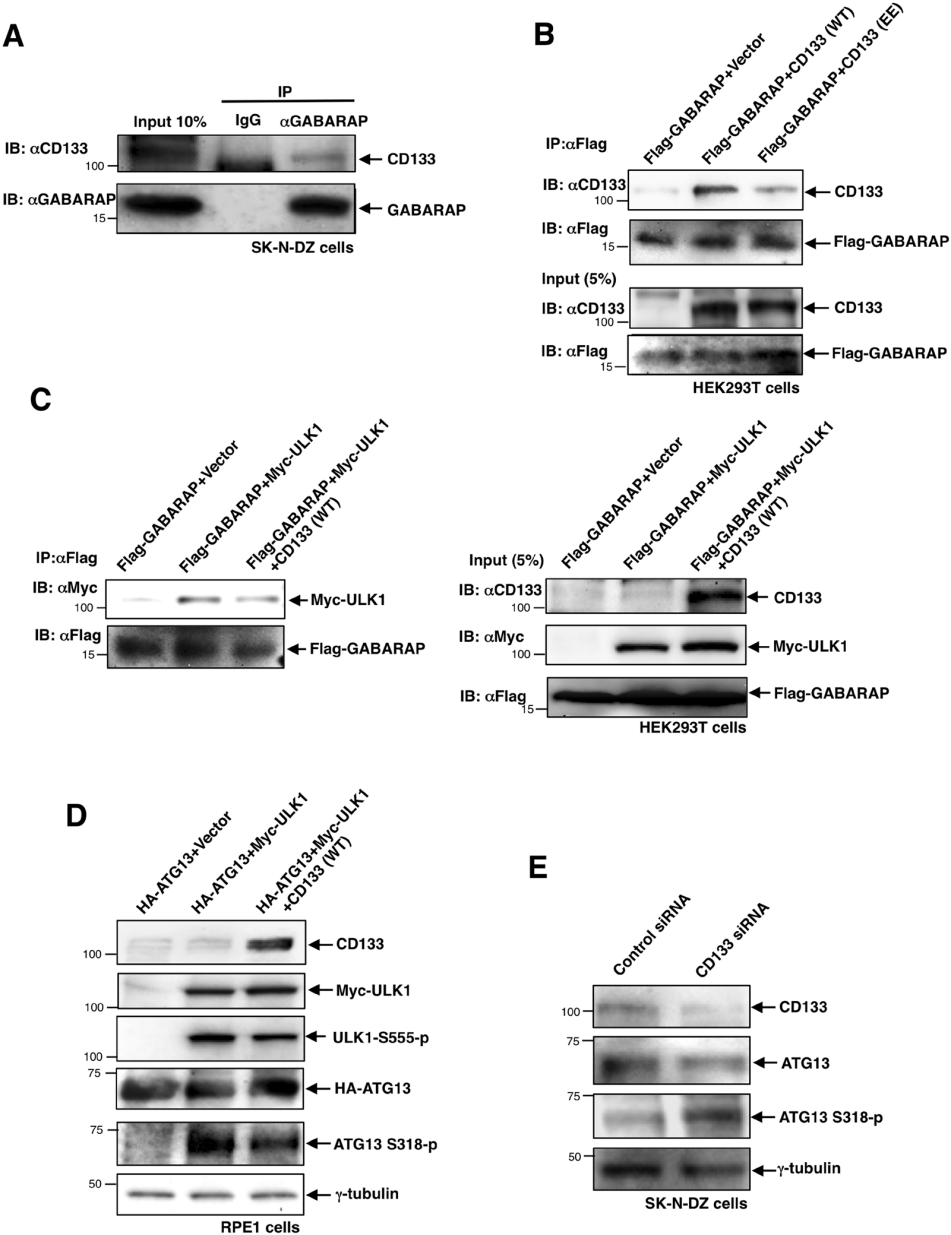
Pericentrosomal CD133 captures GABARAP at the centrosome, thereby inhibiting GABARAP-mediated ULK1 activation. (**A**) Endogenous CD133 interacts with GABARAP in SK-N-DZ cells. Five hundred micrograms of the cell lysate from SK-N-DZ cells was immunoprecipitated with an anti-GABARAP antibody. As a control, immunoprecipitates with preimmune mouse immunoglobulin-G (IgG) were used. Immunoprecipitates were then subjected to immunoblotting using an anti-CD133 antibody. (**B**) GABARAP interacts with CD133 (WT), but not CD133 (EE). Five hundred micrograms of the cell lysate from HEK293T cells transfected with the indicated vectors was immunoprecipitated with an anti-Flag antibody. Immunoprecipitates were then subjected to immunoblotting using an anti-CD133 antibody. The 5% input was also subjected to immunoblotting using anti-CD133 and anti-Flag antibodies. (**C**) The interaction between GABARAP and ULK1 was inhibited by CD133. Five hundred micrograms of the cell lysate from HEK293T cells transfected with the indicated expression vectors was immunoprecipitated with an anti-Flag antibody (left). Immunoprecipitates were then subjected to immunoblotting using an anti-Myc antibody (left). The 5% input was also subjected to immunoblotting using anti-CD133, anti-Myc, and anti-Flag antibodies (right). (**D**) CD133 inhibits the ULK1-mediated phosphorylation of ATG13. Immunoblot of CD133, Myc-ULK1, ULK1S555-p, HA-ATG13, and ATG13-S318-p in RPE1 cells transfected with the expression vector indicated above each lane. An immunoblot of γ-tubulin served as a loading control. (**E**) The knockdown of CD133 enhances the ULK1-mediated phosphorylation of ATG13 in SK-N-DZ cells. Immunoblot of CD133, ATG13, and ATG13-S318-p in SK-N-DZ cells transfected with CD133 siRNA or its control. An immunoblot of γ-tubulin served as a loading control.

We next investigated whether CD133 interacts with GABARAP. The cell lysate from SK-N-DZ cells was immunoprecipitated using an anti-GABARAP antibody, and immunoprecipitates were then blotted using an anti-CD133 antibody. As expected, the CD133 band was detected in the immunoprecipitates (Fig. 7A), indicating the interaction of endogenous CD133 with GABARAP in SK-N-DZ cells. We also examined whether the phosphorylation status of CD133 affects the interaction of CD133 with GABARAP. We co-transfected Flag-GABARAP with CD133 (WT) or CD133 (EE) into HEK293T cells, and performed an immunoprecipitation experiment. The results obtained showed that CD133 (WT), but not CD133 (EE) preferentially interacted with GABARAP (Fig. 7B), suggesting that the Src-mediated phosphorylation form of CD133 shows low affinity for binding GABARAP, and that the unphosphorylated form of CD133 is preferentially recruited to the pericentrosomal region through endocytosis, thereby capturing GABARAP at centrosomes.

We investigated whether CD133 interferes with the GABARAP-ULK1 interaction. We co-transfected the Flag-GABARAP vector and Myc-ULK1 vector with or without the CD133 (WT)-expressing vector into HEK293T cells, and then performed an immunoprecipitation experiment (Fig. 7C). As expected, the GABARAP-ULK1 interaction was partially interrupted in the presence of CD133 expression (Fig. 7C). We subsequently tested whether CD133 interferes with the GABARAP-ULK1 interaction, thereby inhibiting ULK1 kinase activity. We co-transfected the HA-tagged ATG13 (as a substrate for ULK1) vector and Myc-tagged ULK1 vector with or without the CD133 (WT)-expressing vector into RPE1 cells, and then performed an immunoblotting experiment using an anti-phospho-ATG13 antibody. As expected, ULK1 kinase activity decreased in the presence of CD133 expression (Fig. 7D). In contrast, when CD133 expression was knocked down with CD133 siRNA in SK-N-DZ cells, endogenous phospho-ATG13 band intensity increased (Fig. 7E). These results suggest that CD133 inhibits ULK1 kinase activity by capturing GABARAP at centrosomes. Thus, pericentrosomal CD133 captures GABARAP at centrosomes, thereby inhibiting GABARAP-mediated ULK1 activation.

### Pericentrosomal CD133 suppresses cell differentiation by inhibiting autophagy

**Figure 8:**
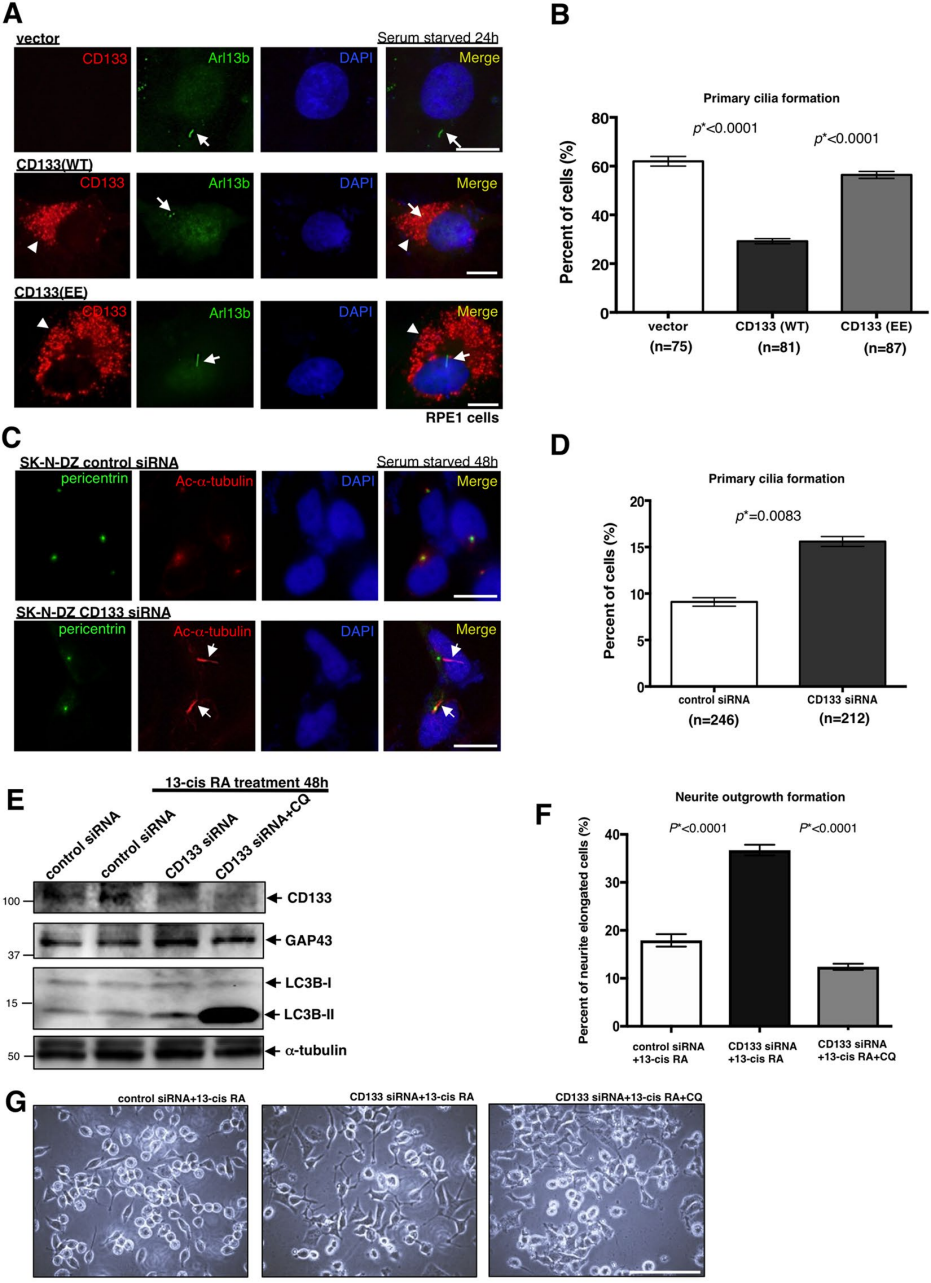
Pericentrosomal CD133 suppresses cell differentiation by inhibiting autophagy. (**A**) Representative images of primary cilium formation in RPE1 cells transfected with the CD133 (WT) or CD133 (EE) expression vector, followed by serum starvation for 24 h. CD133 is red, Arl13b (cilium) is green, and DAPI (DNA) is blue. The arrow shows a primary cilium. Arrowheads show the localisation of CD133. Scale bars, 10 μm. (**B**) Quantification of cells with primary cilia shown in (**A**); mean ± standard error of the mean (SEM) from three experiments, *p < 0.0001, *p < 0.0001, respectively. (**C**) Representative images of primary cilium formation in SK-N-DZ cells transfected with CD133 siRNA or its control, followed by serum starvation for 48 h. Pericentrin (centrosome) is green, acetylated-α-tubulin (cilium) is red, and DAPI (DNA) is blue. Arrows show primary cilia. Scale bars, 10 μm. (**D**) Quantification of cells with primary cilia shown in (**C**); mean ± standard error of the mean (SEM) from three experiments, *p = 0.0083. (**E**) Immunoblot of CD133, GAP43 (axon regeneration marker), and LC3B in SK-N-DZ cells transfected with CD133 siRNA or its control, followed by a 13-cis retinoic acid (RA) treatment for 48 h with or without chloroquine (CQ: autophagy inhibitor). An immunoblot of α-tubulin served as a loading control. (**F**) Representative images of neurite outgrowth in SK-N-DZ cells transfected with CD133 siRNA or its control, followed by a 13-cis RA treatment for 48 h with or without CQ. Scale bars, 100 μm. (**G**) Quantification of cells with neurite elongation shown in (**F**); mean ± standard error of the mean (SEM) from three experiments, *p < 0.0001, *p < 0.0001, respectively.

We investigated the physiological role of pericentrosomal CD133 in the inhibition of autophagy. Primary cilium formation is prominent in RPE1 cells exposed to serum starvation (Supplementary Fig. S5A,B). Autophagic activity is known to be involved in primary cilium formation^28^. Primary cilia are sensory “antenna”-like organelles that survey the outer cell environment. While many normal differentiated cells have cilia, malignant tumours do not. Therefore, we performed a cilium formation assay using RPE1 cells transfected with the CD133 (WT) or CD133 (EE) vector. We found that while CD133 (WT) was recruited to the pericentrosomal region and suppressed cilium formation (Fig. 8A,B), CD133 (EE) was not and did not suppress cilium formation because phosphorylated CD133 did not approach centrosomes (Fig. 8A,B and Supplementary Fig. S5C). In order to elucidate the underlying mechanisms in more detail, we co-transfected Flag-HDAC6 with CD133 (WT), CD133 (EE) or CD133 (FF) into HEK293T cells, and then performed an immunoprecipitation experiment. The results obtained showed that CD133 (WT) and CD133 (FF), but not CD133 (EE) interacted with HDAC6 (Supplementary Fig. S5D). Therefore, the CD133 (WT)-HDAC6 complex interacted with dynein and may have then been effectively transported to the pericentrosomal region to suppress autophagic flux. On the other hand, CD133 (EE) did not form a complex with HDAC6, and, thus, was not transported to the pericentrosomal region to suppress autophagy.

Conversely, CD133 expression was knocked down with siRNA in SK-N-DZ cells, and serum starvation was conducted for 48 h. As expected, primary cilium formation significantly increased in CD133-knockdown SK-N-DZ cells (Fig. 8C,D and Supplementary Fig. S6A). When CD133 expression was also knocked down with siRNA in Huh-7 cells, similar primary cilium formation was detected (Supplementary Fig. S6B,C).

A recent study reported that during primary ciliogenesis, autophagic activity removes centrosomal OFD1 from centriolar satellites^27^. Therefore, we investigated whether pericentrosomal CD133 affects the removal of OFD1 from centriolar satellites during serum starvation. CD133 expression was knocked down with siRNA in SK-N-DZ cells, and serum starvation was conducted for 48 h. As expected, the ratio of removal of OFD1 from centriolar satellites was significantly higher in CD133-knockdown SK-N-DZ cells than in control cells (Supplementary Fig. S6D,E). Thus, pericentrosomal CD133 directly suppresses autophagy flux around centrosomes.

A previous study reported that autophagic activity is necessary for neurite outgrowth in mouse neuroblastoma cells^40^. Therefore, we performed a neurite outgrowth assay using SK-N-DZ cells. CD133 expression was knocked down with siRNA in SK-N-DZ cells, and 13-cis retinoic acid (13-cis RA) was then added to the cells because 13-cis-RA is known to induce the arrest of cell growth and morphological differentiation of human neuroblastoma cell lines^41^. Chloroquine (CQ), an autophagy inhibitor, was also added to knockdown cells treated with 13-cis RA. After 48 h, neurite outgrowth was investigated. We found that while neurite outgrowth significantly increased in CD133-knockdown cells treated with 13-cis RA (Fig. 8E–G), the CQ treatment abolished neurite outgrowth in CD133-knockdown cells treated with 13-cis RA (Fig. 8E–G).

Thus, pericentrosomal CD133 suppresses cell differentiation, such as primary cilium formation and neurite outgrowth, by inhibiting autophagy.

## Discussion

**Figure 9:**
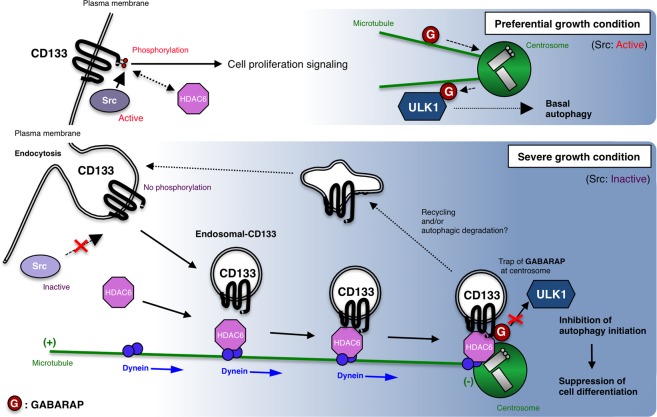
Schematic image of CD133 functions in cells. Under preferential growth (high nutrients) conditions, CD133 is phosphorylated in its intracellular C-terminal domain by Src family tyrosine kinases. As a result, phosphorylated CD133 activates a p85 subunit of phosphoinositide 3-kinase (PI-3K) by binding, and PI-3K, in turn, activates downstream targets, such as Akt, thereby promoting cell proliferation. Autophagic activity is at its basal level. On the other hand, under severe growth (low nutrients) conditions, non-phosphorylated CD133 is transported from the plasma membrane to the pericentrosomal region through endocytosis. After endocytosis, HDAC6 and dynein motors assist CD133 endosome trafficking along microtubules to centrosomes. Endosomal CD133 localised in a pericentrosomal region captures GABARAP to inhibit the GABARAP-ULK1 interaction, thereby suppressing the initiation of autophagy. Endosomal CD133 is a recycling endosome that may be mediated through the functions of Rab11. Thus, pericentrosomal CD133 has the unique property of maintaining a cell undifferentiated status by inhibiting autophagy.

The present results demonstrated that the unphosphorylated form of CD133 is preferentially processed in endosomes, and is then recruited to the pericentrosomal region via an association with the HDAC6-dynein traffic system. Although the transportation of internalized membranous CD133 to endosomes has already been reported^9,20^, the trafficking pathway and functional roles of cytoplasmic CD133 currently remain unknown. We herein revealed for the first time the precise mechanisms of the endosomal CD133 transportation pathway (Fig. 9). Cytoplasmic CD133 expression has been identified as a high-risk factor for survival in HCC^30,42^, ovarian cancer^31^, and glioblastoma^43^. Sasaki *et al*. examined 136 HCC patients and detected the cytoplasmic and plasma membranous types of CD133 expression in 22 (16.2%) and 20 (14.7%) patients, respectively^30^. Thus, cytoplasmic CD133 expression may not be rare in human cancers. Sasaki *et al*. also divided CD133-positive cases into three groups: the plasma membranous type, diffuse cytoplasmic type, and perinuclear dot-like type. We speculate that this perinuclear dot-like type may be due to pericentrosomal CD133 localisation. Therefore, CD133 may have important biological functions at pericentrosomal regions as well as at the plasma membrane.

CD133 was recently shown to positively regulate autophagy for cell survival in the normal retinal pigment epithelium^21^, HCC^15^, and gliomas^14^ under several nutrient-deprived microenvironments. Collectively, these findings show that low nutrient conditions promote the release of CD133 from the plasma membrane to the cytoplasm, and then CD133 also partially co-localises with autophagy-related proteins such as LC3, Beclin, p62, ATG5, and lysosomes upon starvation^14,15,21^. These findings also suggest that Src family kinases, including Src and/or Lyn, are inactivated under these conditions, and the incompletely phosphorylated form of CD133, which cannot interact with HDAC6, accumulates in the cytoplasm, but not the pericentrosomal region. In addition, the incompletely phosphorylated form of CD133 may be preferentially degraded by autophagy. Alternatively, since centrosomes are small organelles, a large amount of overexpressed CD133, which cannot be contained in centrosomes, may be preferentially degraded by autophagy.

The present results revealed that endosomal CD133 does not merely undergo degradation; it also functions as an autophagy inhibitor at the pericentrosomal region (Fig. 9). This result provides a unique insight into the functional relationship among endosomes, centrosomes, and autophagy. A previous study reported that the centrosome regulates the Rab11-dependent recycling endosome pathway at appendages of the mother centriole^44^. Therefore, CD133-positive recycling endosomes may also interact with the mother centriole. An important autophagy factor, GABARAP, was recently shown to localise to centrosomes and play a critical role in the activation of ULK1 kinase and autophagy^38,39^. The present results revealed an inhibitory role for CD133 in GABARAP-mediated autophagy initiation. In fact, the CD133 amino-acid sequence (828–831a. a. (Y-D-D-V)) including the phosphorylation site (Y828) by Src kinase is also conserved as an LC3B-interacting region (LIR: Y/F-X-X-V), which is alternatively recognized as a GABARAP-interacting motif^45^. Therefore, it is possible that CD133 interacts with GABARAP via this potential LIR.

Autophagy also serves as a critical mechanism for stem cells^22^. Autophagic activity is necessary for cell differentiation in NSCs and ESCs^23^. Moreover, autophagy contributes to the maintenance of a stable differentiation state in hepatocytes^46^. In the present study, the knockdown of CD133 significantly induced primary cilium formation in human neuroblastoma and HCC cells. Autophagic activity has been shown to regulate primary ciliogenesis by controlling ciliary protein levels^26–28^. In the present study, we also demonstrated that the knockdown of CD133 with a 13-cis RA treatment significantly induced neurite outgrowth, and that the addition of CQ to CD133 knockdown cells with a 13-cis RA treatment inhibited neurite outgrowth in human neuroblastoma cells. These results indicate that autophagic activity is also necessary for the neural differentiation of human neuroblastoma cells. Thus, our results revealed that pericentrosomal CD133 inhibits autophagic activity in order to maintain the undifferentiated state of neuroblastoma cells. However, since CD133 is preferentially expressed in specific cells, such as neural and hematopoietic stem cells and cancer stem cells, the endosomal CD133-mediated inhibition of autophagy may only occur in specialized cells, and not in all cell types.

In summary, our results provide evidence for pericentrosomal CD133 having the unique property of maintaining the undifferentiated status of cells by inhibiting autophagy.

## Materials and Methods

### Cell culture and transfection

All cell lines (SK-N-DZ (CRL-2149), Caco-2 (HTB-37), RPE1 (CRL-4000), and HEK293T cells (CRL-11268) except Huh-7 were obtained from American Type Culture Collection (ATCC). Huh-7 (JCRB0403) was obtained from Japan Cell Resource Bank (JCRB). All cell lines have been validated by Short Tandem Repeat analysis. These cells were maintained in complete medium [Dulbecco’s modified Eagle’s medium, supplemented with 10% fetal bovine serum, antibiotic-antimycotic solution (Gibco, 15240-096)] in an atmosphere containing 5% CO_2_ at 37 °C. Nocodazole (M1404, final 20 μg/ml for 15 min.), Cycloheximide (C4859, final 50 μg/ml) and Monensin (M5273, final 20 μM) were obtained from Sigma-Aldrich. Ciliobrevin D (250401, final 50 μM for 1 h) and Pitstop2 (ab120867, final 30 μM for 1 h) were obtained from Merk and Abcam, respectively. Dasatinib (11498, final 5 μM for 5 h in SK-N-DZ and final 15 μM for 20 h in Caco-2 cells) were purchased from Cayman Chemicals. 13-cis-retinoic acid (RA) (R0088, final 25 μM for 48 h) were obtained from Tokyo Chemical Industry. Finally, Chloroquine (038–1791, final 20 μM) were purchased from Wako, Inc.. Lipofectamine 3000 was used for plasmids transient transfection according to the manufacturer’s instructions. Cells were analysed 20 h after transfection. For RNA interference, siRNA duplexes were transfected using Lipofectamine RNAiMAX according to the manufacturer’s instructions. All siRNAs (control siRNA (AM4611), HDAC6 siRNA (AM5133), CD133 siRNA (4392420), and Src siRNA (S13414)) were obtained from ThermoFisher Scientific.

### Plasmids

The pCMV-Flag vector (PS100001) and pCMV-Flag-HDAC6 vector (RC209649) were obtained from OriGene Technologies. The pcDNA 3.1 (V79020) was purchased from ThermoFisher Scientific. Full length CD133, Src, and GABARAP cDNAs were purchased from GenScript. The cDNA was confirmed by DNA sequencing. Subsequently, We cloned these cDNAs into pcDNA expression vector, respectively. For constructing Src (DN), CD133 (EE), and CD133 (FF) plasmids, QuickChange site-directed mutagenesis kit (#200519-5: Agilent Technologies) were used. All mutant vectors were confirmed by DNA sequencing. Myc-hULK1 vector (#31961)^47^ and HA-hATG13 (#31967)^47^ were obtained from Addgene.

### Antibodies

The primary antibodies used were as follows: anti-CD133 (AC133) (1:10 for immunofluorescence (IF), 1:100 for immunoblotting (WB) and 5 μg/500 μg of cell lysates for immunoprecipitation (IP), 130-090-422, Miltenyi Biotec), anti-CD133 (W6B3C1) (1:100 for WB, 130-092-395, Miltenyi Biotec), anti-CD133 (1:100 for IF, ab19898, abcam), anti-CD133 (1:50 for IF, #64326, Cell Signaling Technology), anti-α-tubulin (DM1A) (1:1000 for WB, T9026, Sigma-Aldrich), anti-γ-tubulin (GTU88) (1:200 for IF and 1:1000 for WB, T6557, Sigma-Aldrich), anti-Rab11 (1:100 for IF, #5589, Cell Signaling Technology), anti-pericentrin (1:250 for IF, NBP1-8771, Novus), anti-GM130 (1:100 for IF, PM1061, MBL), anti-LAMP-2 (1:100 for IF, GTX103214, GeneTex), anti-Src (1:100 for IF and 1:500 for WB, #2109, Cell Signaling Technology), anti-Src-Y418-p (1:50 for IF and 1:100 for WB, #2101, Cell Signaling Technology), anti-LC3B (1:200 for IF and 1:500 for WB, GTX127375, GeneTex), anti-GABARAP (1:100 for IF and 1:400 for WB, AP1821a, Abgent), anti-GABARAP (5 μg/500 μg of cell lysates for IP, sc-377300, Santa Cruz Biotechnology), anti-Flag (5 μg/500 μg of cell lysates for IP, F3165, Sigma Aldrich), anti-Flag (1:100 for IF, 1:500 for WB, #14793, Cell Signaling Technology), anti-Myc (4E10) (5 μg/500 μg of cell lysates for IP, 1:500 for WB, 017-21871, Wako), anti-HA (1:500 for WB, 014-21881, Wako), anti-GAP43 (1: 400 for WB, ab75810, Abcam), anti-Arl13b (1:100 for IF, 17711-1-AP, Proteintech), anti-acetylated-α-tubulin (1:10000 for IF, T7451, Sigma Aldrich), anti-HDAC6 (1:100 for IF, AP1106, Abgent), anti-HDAC6 (1:500 for WB, sc-11420, Santa Cruz Biotechnology), anti-ATG13 (1:400 for WB, #13273, Cell Signaling Technology), anti-ATG13-S318-p (1:400 for WB, 600-401-C49S, Rockland), anti-ULK1(1:400 for WB, #5869, Cell Signaling Technology), anti-ULK1-S555-p (1:400 for WB, #8054, Cell Signaling Technology), and anti-OFD1 (1:100 for IF, ABC961, Millipore). Horseradish peroxidase-labelled secondary antibodies were purchased from General Electric (GE) Healthcare and used at 1:10000. Fluorescence-labelled Alexa secondary antibodies used in this study were obtained from Molecular Probes and used at 1:500.

### Indirect immunofluorescence and cell imaging

Cells grown on coverslips were briefly washed in Phosphate-buffered saline (PBS) three times, and then fixed with 10% formalin for 20 min at room temperature (RT) or ice cold methanol for 20 min at −20 °C. The cells were treated with 1% NP-40 in PBS solution for 10 min, and were incubated with blocking solution (15% bovine serum albumin (BSA) in PBS) for 1 h. The cells were then probed with primary antibodies for 1 h at 37 °C and antibody-antigen complexes were detected with either Alexa Fluor 594- or Alexa Fluor 488-conjugated donkey secondary antibody by incubation for 1 h at RT. The samples were washed three times with Tris-buffered saline (TBS) after each incubation and then counterstained with 4′, 6′-diamidino-2-phenylindole (DAPI). Immunostained cells were examined under a fluorescence microscope (Olympus IX73, Tokyo, Japan) using a 100x or 60x objective lens. The fluorescence images were captured with a CCD camera (Olympus, DP27) and processed with Adobe Photoshop CS5 and ImageJ.

### Immunohistochemical Staining of Tissue microarray (TMA)

Human neuroblastoma TMA (MC-602) was obtained from Biomax, Inc. Immunohistochemical staining was performed on Human neuroblastoma TMA (The 5 μm thick tissue sections were equipped with duplicate 1.5 mm cores of neuroblastoma tissues from various sites including the retroperitoneum, mediastinum, abdominal and pelvic cavities, and the adrenal glands of 25 patients) using anti-CD133 (AC133) as primary antibody. The TMA sections were immersed in methanol containing 0.3% hydrogen peroxide for 15 min to block endogenous peroxidase activity. Antigens were retrieved by autoclaving for 10 min in citrate buffer (pH 6.0). Sections were then incubated with primary antibody for 1 h at RT. The TMA were then incubated in the Envision system for 30 min at room temperature with the addition of 3, 3-diaminobenzidine (DAB) to achieve visualization of the antigen.

### Immunoelectron microscopy

The samples were fixed with 4% paraformaldehyde (PFA) and 0.1% glutaraldehyde (GA) for 20 min at RT. They were then put into a refrigerator for 1 h in order to lower the temperature at 4 °C, and then they were washed 3 times in PBS for 15 min each. The samples were then dehydrated in graded ethanol solutions (50%, 70%) at 4 °C for 10 min each. The samples were infiltrated with a 50150 mixture of ethanol and resin (LR. white; London Resin Co. Ltd., Berkshire, UK) for 10 min each 3 times. After this infiltration, they require 3 changes of 100% LR white at 4 °C for 20 min each. The samples were transferred to a fresh 100% resin, and were polymerized at 50 °C overnight. The polymerized resins were ultra-thin sectioned at 80 nm with a diamond knife using an ultra-microtome (Ultracut UCT: Leica, Vienna, Austria) and the sections were mounted on nickel grids. The grids were incubated with the primary antibody (mouse anti-CD133 (AC133)) in BSA and PBS at 4 °C overnight. They were then washed with 1% BSA and PBS 3 times for 1 min. They were subsequently incubated with the secondary antibody conjugated to 5 nm gold particles (goat anti-mouse IgG pAb) for 2 h at room temperature. And after washing with PBS, the grids were placed in 2% GA in PBS. After the grids were dried, they were stained with 2% uranyl acetate for 15 min, and with Lead stain solution (Sigma-Aldrich Co., Tokyo, Japan) at RT for 3 min. The grids were observed by a transmission electron microscope (JEM-1400Plus: JEOL Ltd., Tokyo, Japan) at an acceleration voltage of 80 kV. Digital images (2048 × 2048 pixels) were taken with a CCD camera (VELETAZ Olympus).

### Immunoblotting

Cells were lysed in SDS/Nonidet P-40 lysis buffer (1% SDS, 1% Nonidet P-40, 50 mM Tris [pH 8.0], 150 mM NaCl, 2 μg/ml leupeptin, 2 μg/ml aprotinin, 1 mM phenylmethylsulfonyl fluoride [PMSF], 5 mM NaF, 100 μM Na_3_VO_4_). The lysates were boiled for 5 min and then cleared by centrifugation at 15,000 rpm and 4 °C. Protein concentration of the supernatant was determined using a Bradford protein assay reagent (BioRad). The lysates were further boiled for 5 min in sample buffer. Samples were then resolved by SDS-PAGE and transferred onto Immobilon-P (Millipore Corp.) sheets. The blots were first incubated in blocking buffer (5% [w/v] nonfat dry milk in Tris-buffered saline [TBS] plus 0.05% Tween 20) for 1 h. The blots were then incubated with a primary antibody for 16 h at 4 °C, followed by incubation with a horseradish peroxidase-conjugated secondary antibody for 1 h at room temperature. The antibody-antigen complex was visualized by ECL-plus chemiluminescence (GE Healthcare).

### Immunoprecipitation

For immunoprecipitations, cells were lysed in 0.5% Nonidet P-40 lysis buffer. 500 microgram of lysates was pre-cleared by incubation with 20 μl of protein G- or A-conjugated agarose for 1 h at 4 °C and incubated on a platform shaker for 3 h with the primary antibody (5 μg) at 4 °C. Protein G- or A-conjugated agarose (40 μl of protein) was then added to the lysate, and the mixture was further incubated on a platform shaker for 1 h at 4 °C, spun down and washed three times in wash buffer (0.1% Nonidet P-40, 50 mM Tris [pH 8.0], 150 mM NaCl, 2 μg/ml leupeptin, 2 μg/ml aprotinin, 1 mM phenylmethylsulfonyl fluoride [PMSF], 5 mM NaF, 100 μM Na3VO4). After these washes, proteins bound to the beads were eluted with sample buffer by boiling for 5 min, separated by 12% or 10% SDS-PAGE and analysed by immunoblotting.

### Quantification and statistical analysis

All statistical analyses were done using Graphpad Prism 7. Experimental groups were compared using unpaired Student’s *t* test, owing to the binary nature of the data sets. Probability values less than 0.05 were considered significant, and data are represented as mean ± standard error of the mean (SEM).

## Supplementary information

Supplementary info

## Data Availability

The datasets generated during and/or analysed during the current study are available from the corresponding author on reasonable request.
