# Supplementary material for: Recycling endosomal CD133 functions as an inhibitor of autophagy at the pericentrosomal region
Source: Sci Rep. 2019 Feb 19;9:2236. doi: 10.1038/s41598-019-39229-8 (PMC6381095; doi:10.1038/s41598-019-39229-8)
Supplement: Supplementary file 1 — Supplementary info [file 41598_2019_39229_MOESM1_ESM.pdf]

Supplementary information

Recycling endosomal CD133 functions as an inhibitor of autophagy at the pericentrosomal region

Hideki Izumi, Yuanyuan Li, Masami Shibaki, Daisuke Mori, Michio Yasunami, Seiji Sato, Hisashi Matsunaga, Takao Mae, Kenji Kodama, Takehiko Kamijo, Yasuhiko Kaneko, and Akira Nakagawara

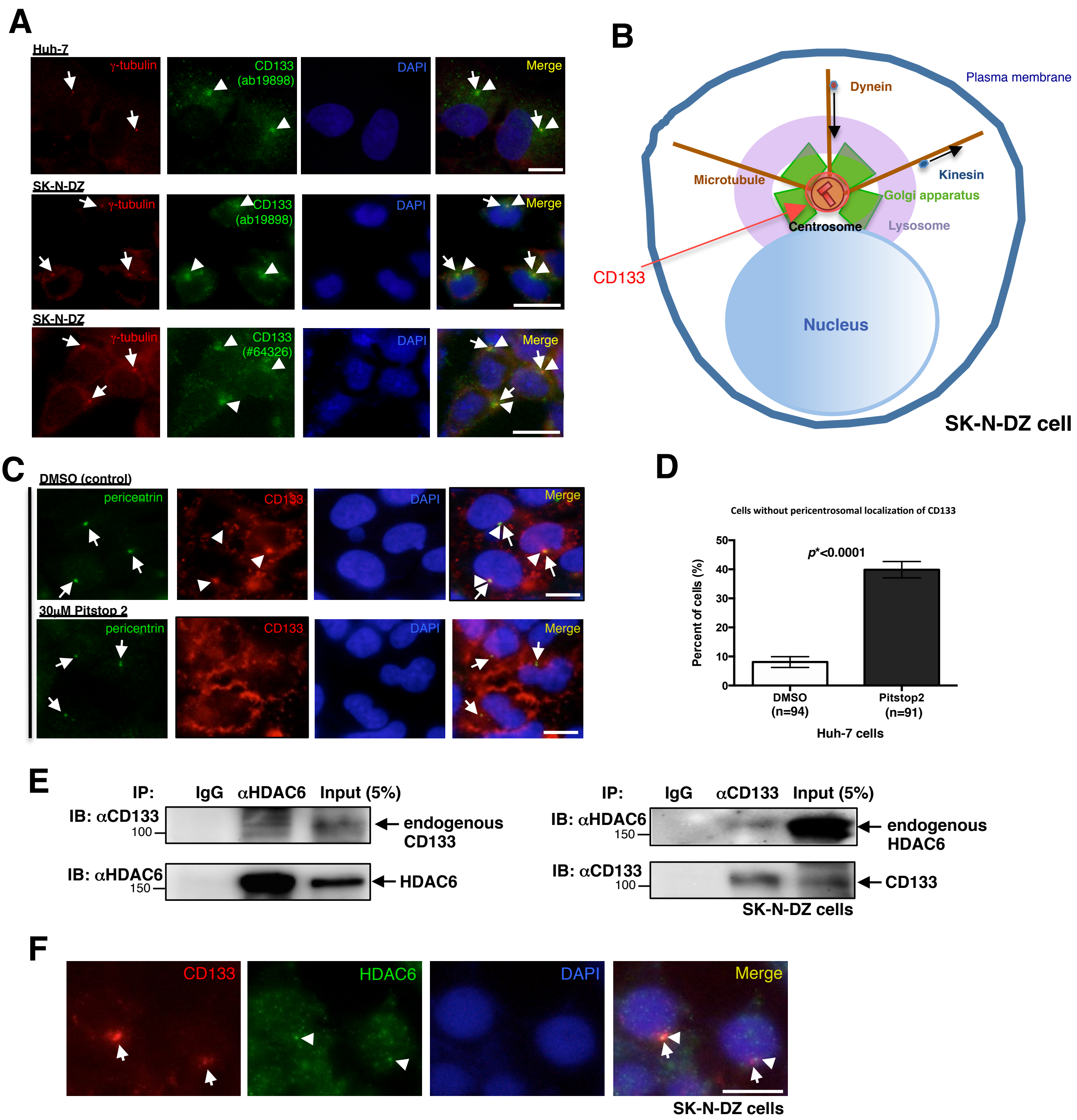

Supplementary Figure S1 Membranous CD133 is transported to the pericentrosomal region via the dynein-based traffic system. **(A)** Representative immunostaining images of the subcellular localisation of CD133 in Huh-7 and SK-N-DZ cells by using anti-CD133 (abcam, ab19898) and anti-CD133 (Cell Signaling Technology, #64326) antibodies. **(B)** Highlighted image of the subcellular localisation of centrosomes, the Golgi apparatus, and lysosomes in SK-N-DZ cells. **(C)** The treatment of the endocytosis inhibitor, Pitstop 2, causes the plasma membranous localization of CD133 in Huh-7 cells. 0.1% DMSO-treated cells are used as a control. Pericentrin is green, CD133 is red, and DAPI (DNA) is blue. Arrows show centrosomes. Arrowheads show CD133 signals at the pericentrosomal region. **(D)** Quantification of cells with the cell peripheral localisation near the plasma membrane of CD133 shown in **(C)**; mean ± standard error of the mean (SEM) from three experiments, $p^*<0.0001$. **(E)** Endogenous CD133 interacts with HDAC6 in SK-N-DZ cells. Five hundred micrograms of the cell lysate from SK-N-DZ cells was immunoprecipitated with an anti-HDAC6 antibody. As a control,immunoprecipitates with preimmune rabbit immunoglobulin-G (IgG) were used. Immunoprecipitates were then subjected to immunoblotting using an anti-CD133 antibody (the left blot). Conversely, 500 μg of the cell lysate was immunoprecipitated with an anti-CD133 antibody, and immunoprecipitates were subjected to immunoblotting using an anti-HDAC6 antibody. As a control, immunoprecipitates with preimmune mouse immunoglobulin-G (IgG) were used (the right blot). **(F)** Representative images of the CD133-HDAC6 interaction at the pericentrosomal region in SK-N-DZ cells. CD133 is red, HDAC6 is green, and DAPI (DNA) is blue. Arrows show CD133 signals. Arrowheads show pericentrosomal HDAC6 signals. All scale bars, 10 µm.

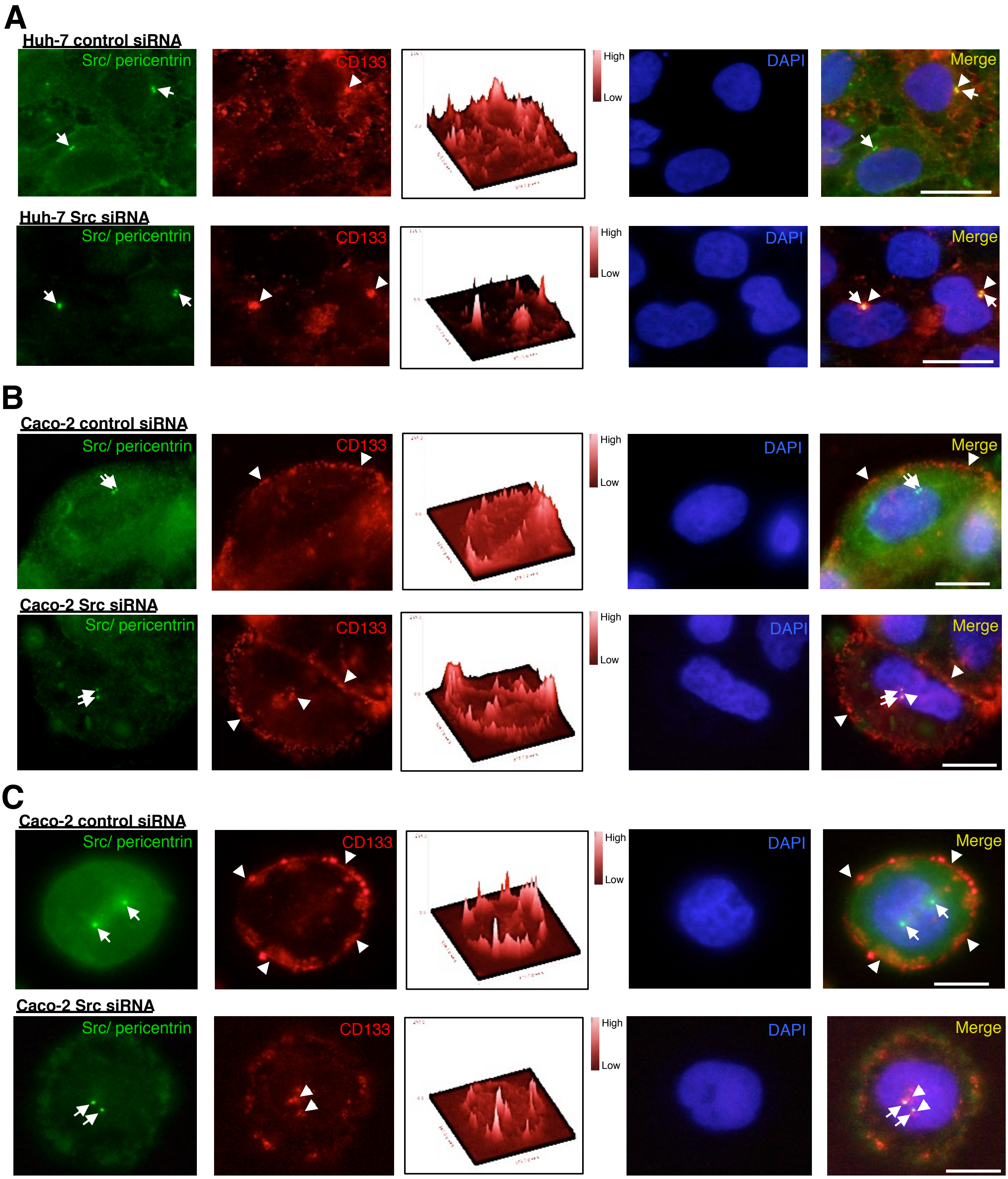

Supplementary Figure S2 Src activity is necessary for the plasma membrane localisation of CD133. **(A)** Representative images of CD133 localised to the pericentrosomal region in Huh-7 cells transfected with Src siRNA or its control. Src and pericentrin are green, CD133 is red, and DAPI (DNA) is blue. Arrows show centrosomes. Arrowheads show CD133 signals at the pericentrosomal region. Note that approximately 12% of Huh-7 cells show originally pericentrosomal localisation of CD133. When Src knockdown was examined, approximately 35% of Huh-7 cells showed pericentrosomal localisation of CD133. The signal intensity of CD133 in each cell is also shown as a three-dimensional figure. **(B)** Representative images of CD133 signals localised to the plasma membrane (control siRNA transfection) or the membranous and pericentrosomal regions (Src siRNA transfection) in Caco-2 cells (flat morphology). Src and pericentrin are green, CD133 is red, and DAPI (DNA) is blue. Arrows show centrosomes. Arrowheads show CD133 signals at the membrane or membranous/pericentrosomal regions. The signal intensity of CD133 in each cell is also shown as a three-dimensional figure. **(C)** Representative images of CD133 signals localised to the plasma membrane (control siRNA transfection) or pericentrosomal region (Src siRNA transfection) in Caco-2 cells (round morphology). Src and pericentrin are green, CD133 is red, and DAPI (DNA) is blue. Arrows show centrosomes, and arrowheads show CD133 signals at the plasma membrane (control siRNA) or pericentrosomal region (Src siRNA). In this experiment, peripheral colony-forming Caco-2 cells were counted. The signal intensity of CD133 in each cell is also shown as a three-dimensional figure. All scale bars, 10 μm.

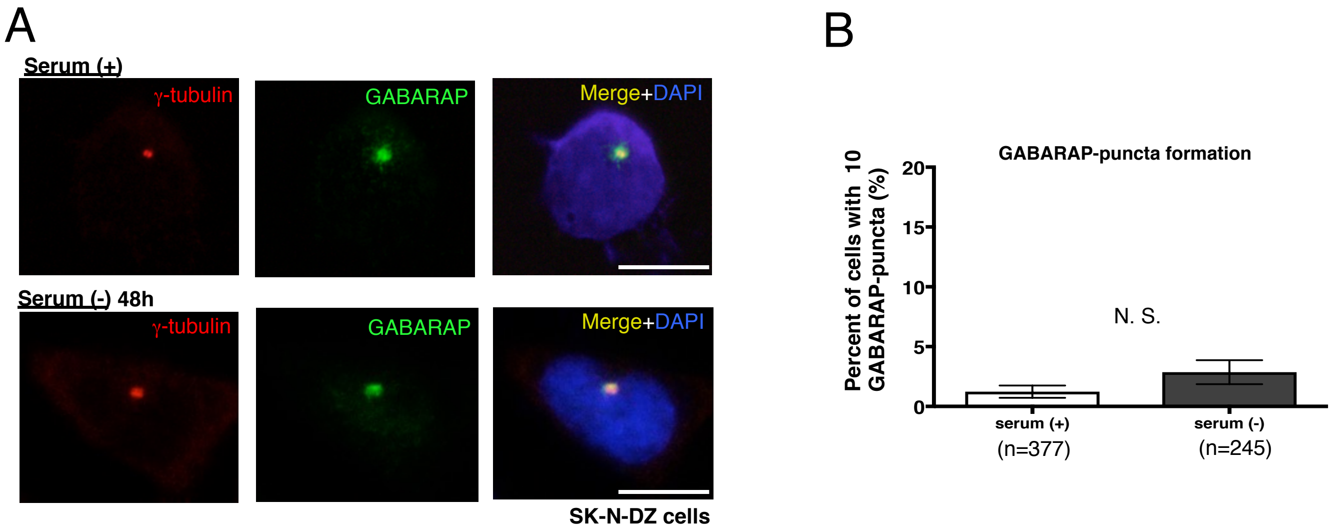

Supplementary Figure S3. Pericentrosomal CD133 suppresses serum starved induced-autophagy in SK-N-DZ cells **(A)** Representative images of centrosomal localisation of GABARAP in SK-N-DZ cells with or without serum starvation for 48 h. γ-Tubulin is red, GABARAP is green, and DAPI (DNA) is blue. Scale bars, 10 μm. **(B)** Quantification of cells with GABARAP-puncta in SK-N-DZ cells with or without serum starvation for 48 h shown in (**A**); mean ± standard error of the mean (SEM) from three experiments, N. S. means not significant.

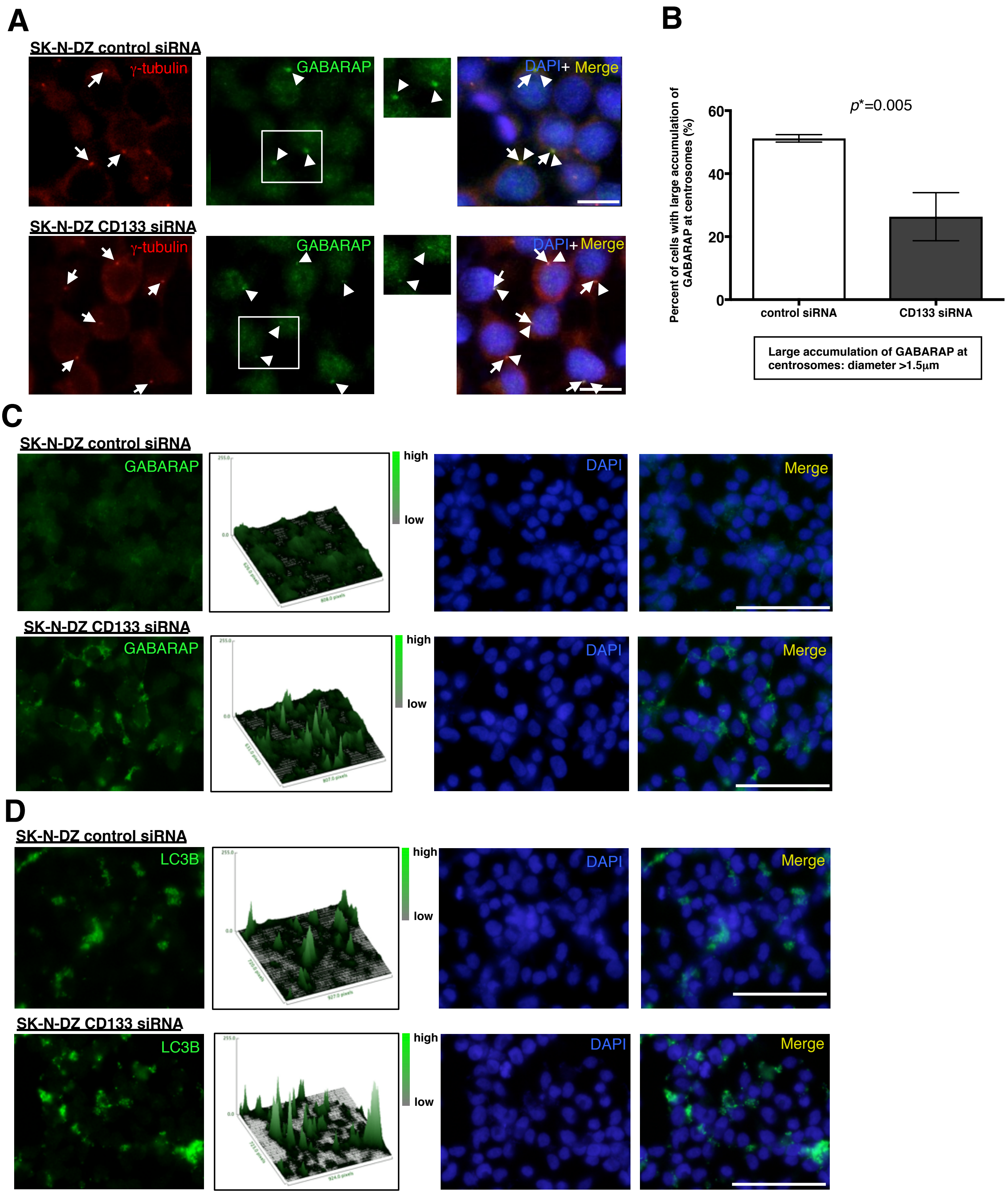

Supplementary Figure S4 Knockdown of CD133 induces autophagy in SK-N-DZ cells. **(A)** The knockdown of CD133 normalizes GABARAP signals from the accumulation type to the non-accumulation type at the centrosome in SK-N-DZ cells. γ-Tubulin is red, GABARAP is green, and DAPI (DNA) is blue. Arrows show centrosomes. Arrowheads show the centrosomal localization of GABARAP. Scale bars, 10 µm. **(B)** Quantification of cells with the accumulation type of GABARAP signals at the centrosome (diameter >1.5 µm) of GABARAP in **(A)**; mean ± standard error of the mean (SEM) from three experiments, p^*=0.005. **(C)** Representative images of GABARAP-puncta during the induction of autophagy (serum starvation for 48h) in SK-N-DZ cells transfected with control or CD133 siRNA. GABARAP is green and DAPI (DNA) is blue. The signal intensity of GABARAP-puncta in each cell is also shown as a three-dimensional figure. Scale bars, 100 µm. **(D)** Representative images of LC3B-puncta during autophagy induction (serum starvation for 48h) in SK-N-DZ cells transfected with control or CD133 siRNA. LC3B is green and DAPI (DNA) is blue. The signal intensity of LC3B-puncta in each cell is also shown as a three-dimensional figure. Scale bars, 100 µm.

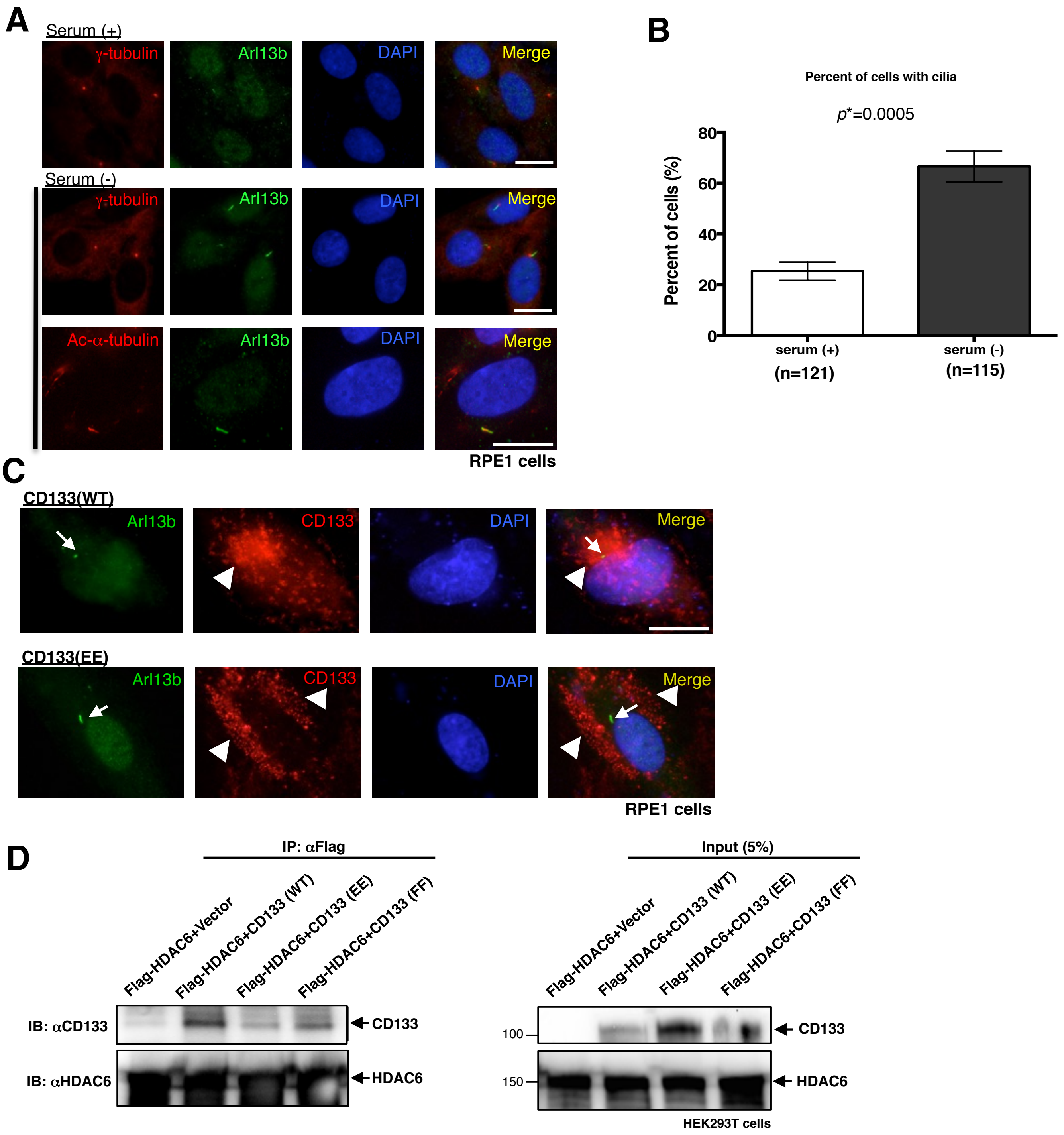

Supplementary Figure S5 Pericentrosomal CD133 suppresses primary ciliogenesis by inhibiting autophagy (1). **(A)** Representative images of primary cilium formation in RPE1 cells with or without serum starvation for 24 h. γ-Tubulin or acetylated-α-tubulin(centrosome)is red, Arl13b (cilium) is green, and DAPI (DNA) is blue. Scale bars, 10 μm. **(B)** Quantification of cells with primary cilia shown in **(A)**; mean ± standard error of the mean (SEM) from three experiments, *p**=0.005. **(C)** Representative images of primary cilium formation in RPE1 cells transfected with the CD133 (WT) or CD133 (EE) expression vector, followed by serum starvation for 24 h. CD133 is red, Arl13b (cilium) is green, and DAPI (DNA) is blue. Arrows show primary cilia. Arrowheads show the distribution pattern of CD133. Scale bars, 10 μm. **(D)** HDAC6 preferentially interacts with CD133 (WT) and CD133 (FF), but not CD133 (EE). Five hundred micrograms of the cell lysate from HEK293T cells transfected with the indicated vectors was immunoprecipitated with an anti-Flag antibody. Immunoprecipitates were then subjected to immunoblotting using an anti-CD133 antibody (left). The 5% input was also subjected to immunoblotting using an anti-CD133 antibody (right).

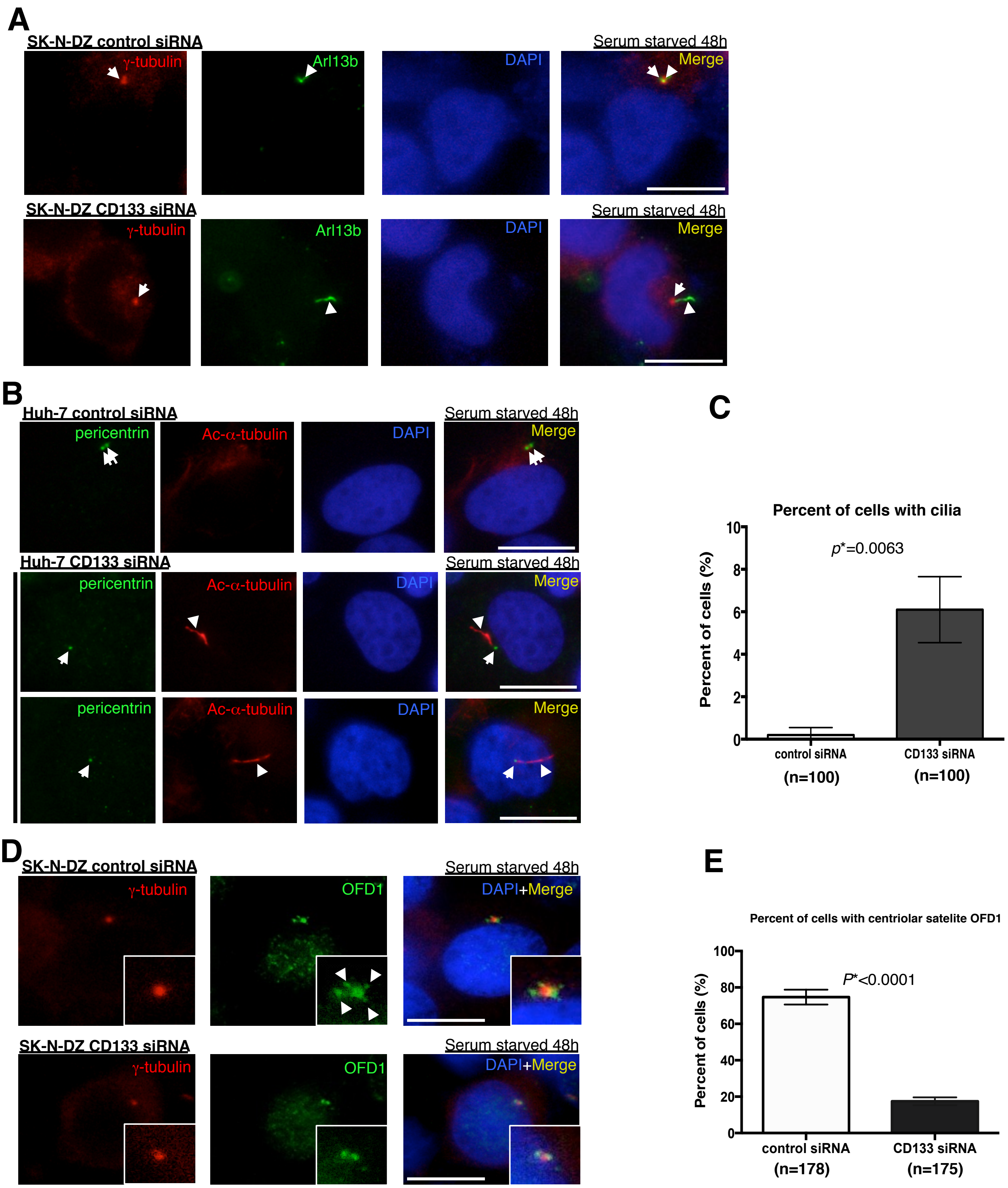

Supplementary Figure S6 Pericentrosomal CD133 suppresses primary ciliogenesis by inhibiting autophagy (2). **(A)** Representative images of primary cilium formation in SK-N-DZ cells transfected with CD133 siRNA or its control, followed by serum starvation for 48 h. γ-Tubulin (centrosome) is red, Arl13b (cilium) is green, and DAPI (DNA) is blue. Arrows show centrosomes. Arrowheads show cilia. Scale bars, 10 μm. **(B)** Representative images of primary cilium formation in Huh-7 cells transfected with CD133 siRNA or its control, followed by serum starvation for 48 h. Pericentrin (centrosome) is green, acetylated α-tubulin (cilium) is red, and DAPI (DNA) is blue. Arrows show centrosomes. Arrowheads show cilium. Scale bars, 10 μm. **(C)** Quantification of CD133 knockdown or control Huh-7 cells with primary cilia shown in **(B)**; mean ± standard error of the mean (SEM) from three experiments, p^*=0.0063. **(D)** Representative images of centriolar satellites in SK-N-DZ cells transfected with CD133 siRNA or its control, followed by serum starvation for 48 h. γ-Tubulin (centrosome) is red, OFD1 (centriole and centriolar satellite) is green, and DAPI (DNA) is blue. Arrowheads show centriolar satellites. Scale bars, 10 μm. **(E)** Quantification of CD133 knockdown or control SK-N-DZ cells with centriolar satellites OFD1 shown in **(D)**; mean ± standard error of the mean (SEM) from three experiments, p^*<0.0001.

Figure 1K

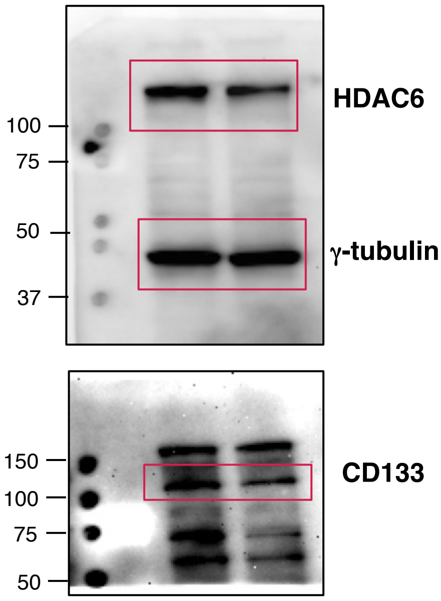

Figure 2B

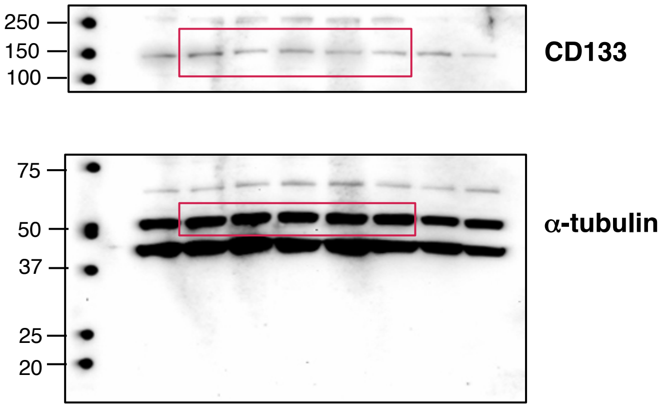

Figure 3B and 3E

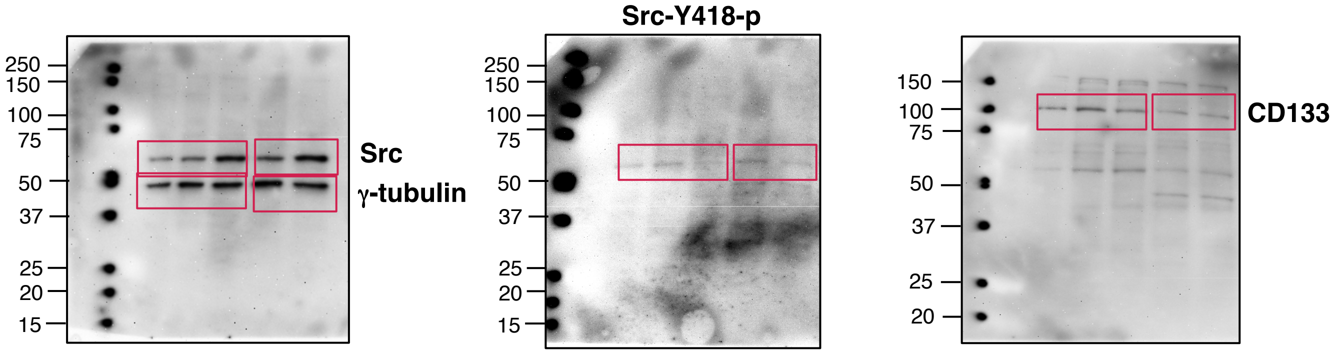

Figure 3H

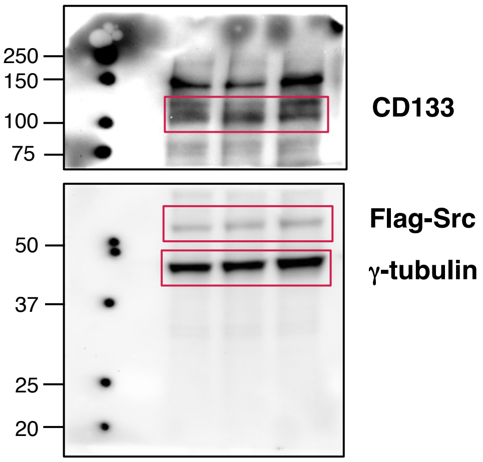

Figure 3J and 3L

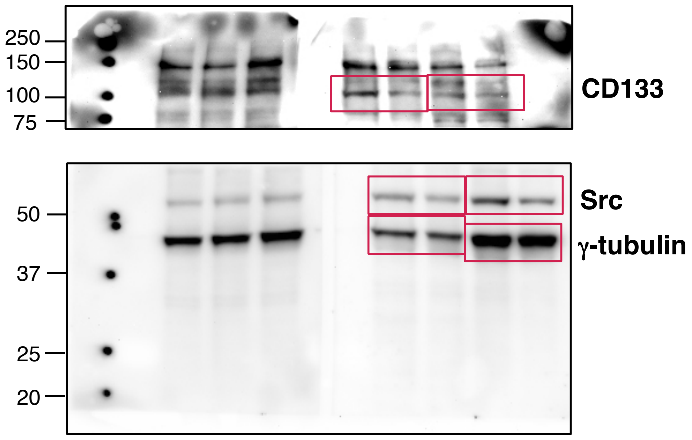

Supplementary Figure S7. Original uncropped scans of immunoblots #1

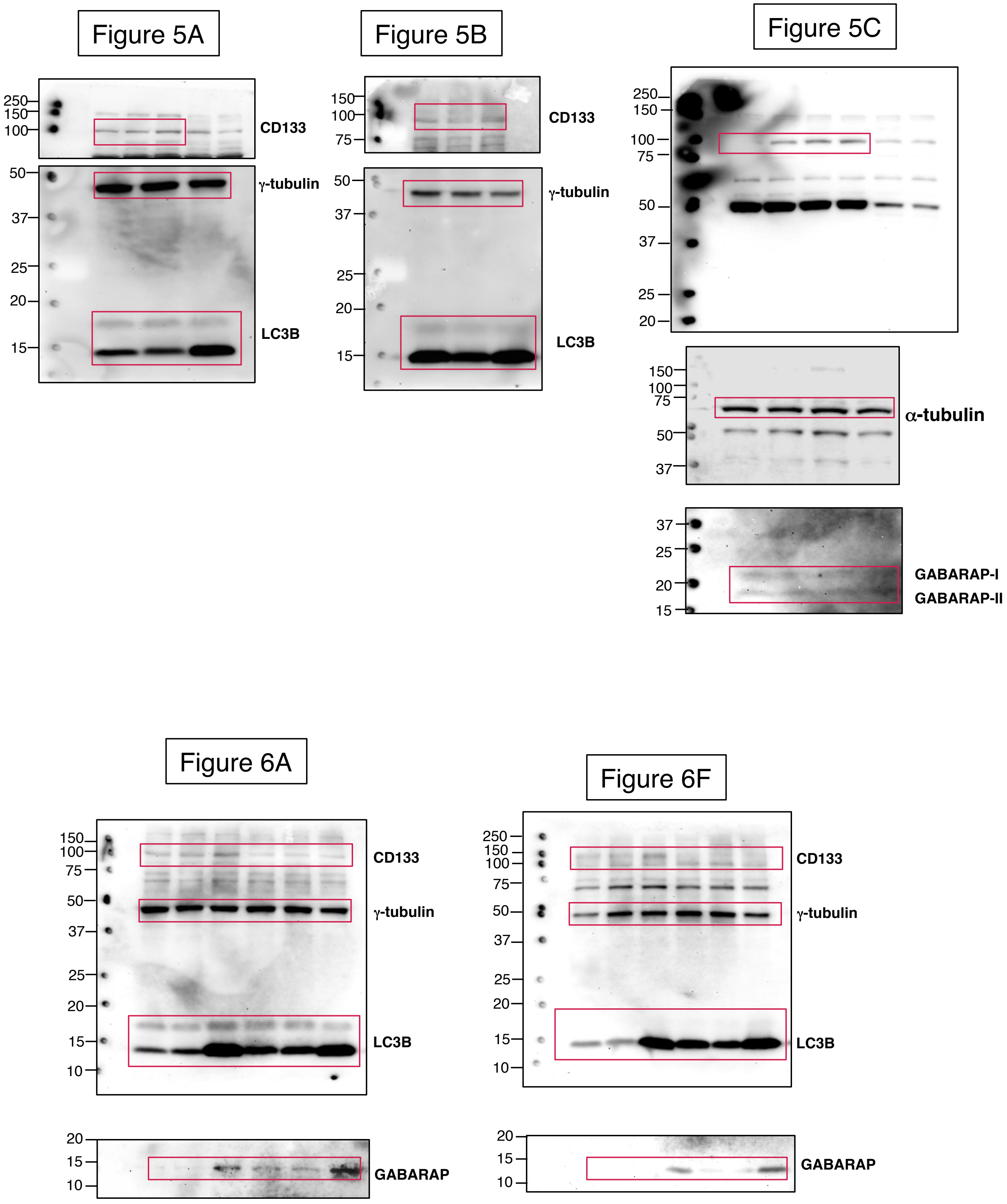

Supplementary Figure S8. Original uncropped scans of immunoblots #2

Figure 8

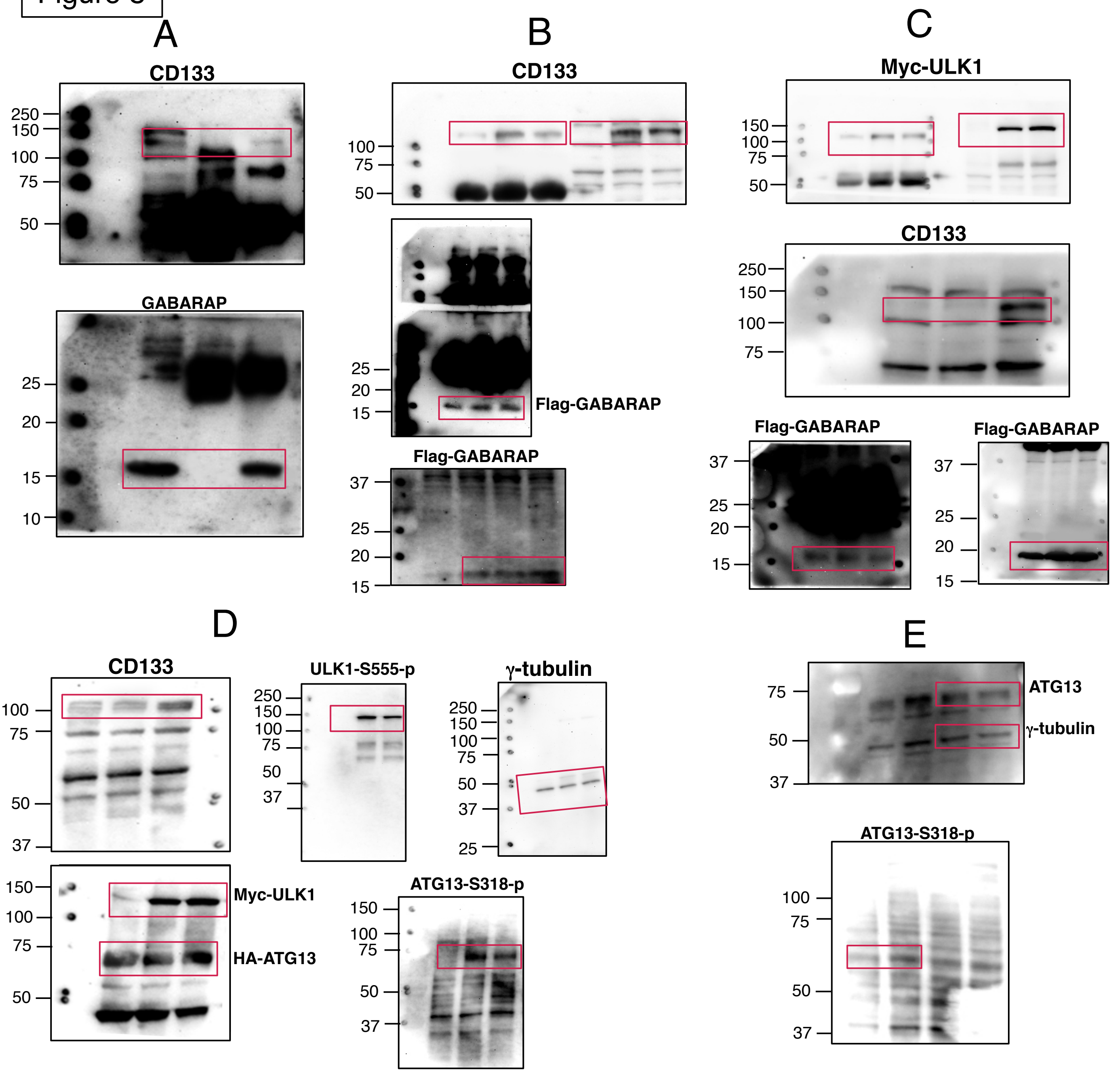

Figure 8E

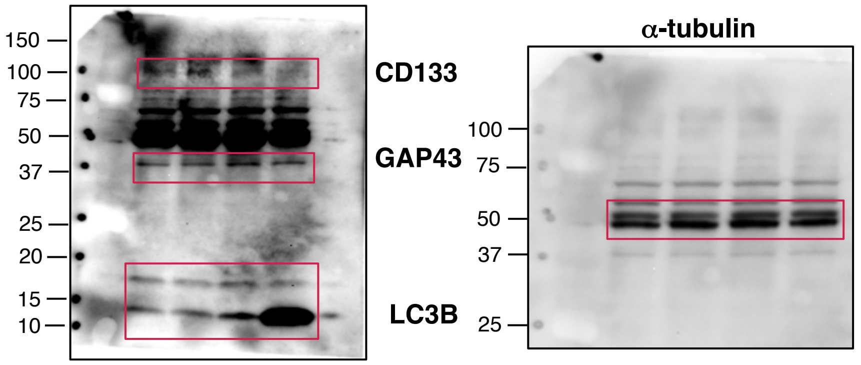

Supplementary Figure S9.
Original uncropped scans of immunoblots #3

Figure S1D

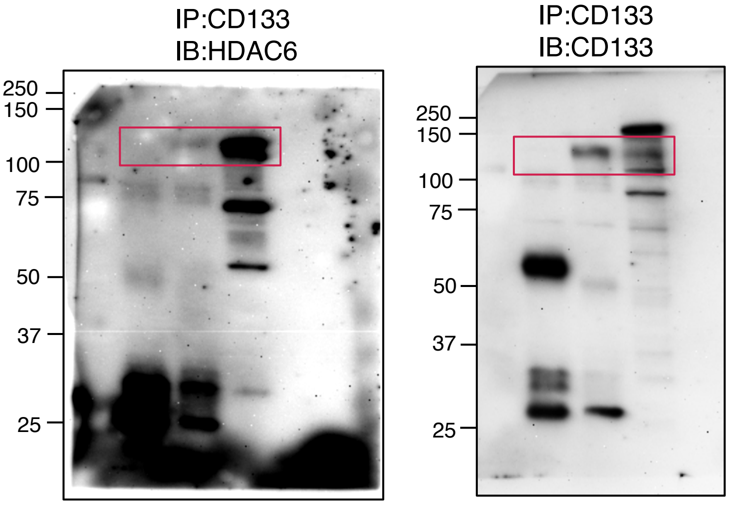

Figure S5D

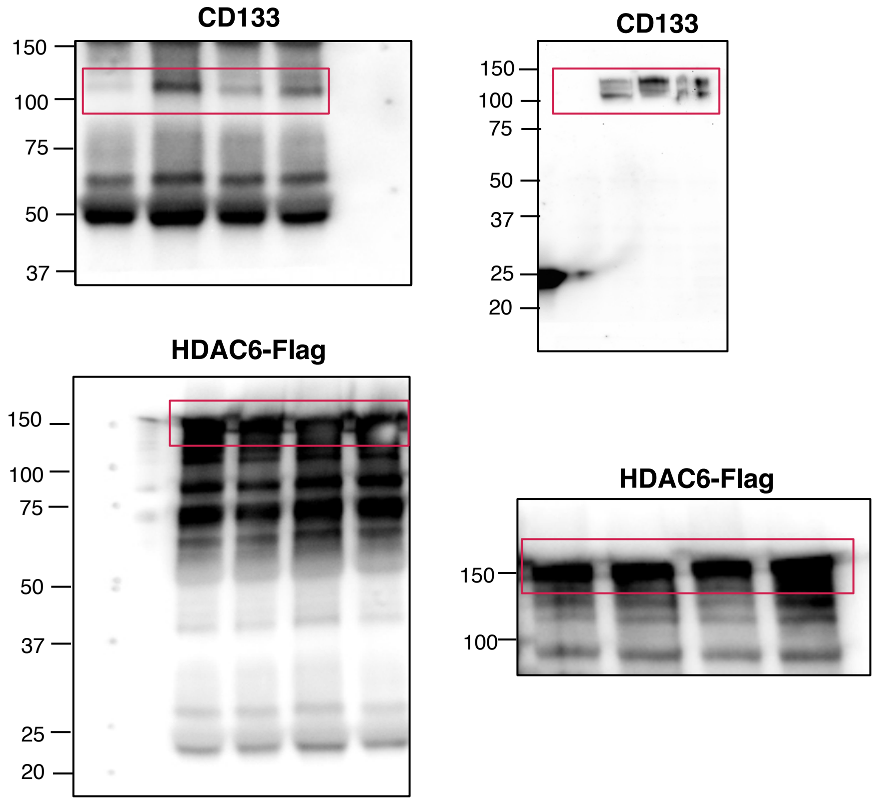

Supplementary Figure S10. Original uncropped scans of immunoblots #4
